# HIV-1 Gag release from yeast reveals ESCRT interaction with the Gag N-terminal protein region

**DOI:** 10.1074/jbc.RA120.014710

**Published:** 2021-01-13

**Authors:** Birgit Meusser, Bettina Purfuerst, Friedrich C. Luft

**Affiliations:** 1 Charité Medical Faculty, Berlin, Germany; 2 Max-Delbrück-Center for Molecular Medicine, Berlin, Germany; 3 Experimental and Clinical Research Center, Berlin, Germany

**Keywords:** Gag, human immunodeficiency virus (HIV), endosomal sorting complexes required for transport (ESCRT), yeast, Saccharomyces cerevisiae, virus release, matrix, ALIX, Bro1, plasma membrane

## Abstract

The HIV-1 protein Gag assembles at the plasma membrane and drives virion budding, assisted by the cellular endosomal complex required for transport (ESCRT) proteins. Two ESCRT proteins, TSG101 and ALIX, bind to the Gag C-terminal p6 peptide. TSG101 binding is important for efficient HIV-1 release, but how ESCRTs contribute to the budding process and how their activity is coordinated with Gag assembly is poorly understood. Yeast, allowing genetic manipulation that is not easily available in human cells, has been used to characterize the cellular ESCRT function. Previous work reported Gag budding from yeast spheroplasts, but Gag release was ESCRT-independent. We developed a yeast model for ESCRT-dependent Gag release. We combined yeast genetics and Gag mutational analysis with Gag-ESCRT binding studies and the characterization of Gag-plasma membrane binding and Gag release. With our system, we identified a previously unknown interaction between ESCRT proteins and the Gag N-terminal protein region. Mutations in the Gag-plasma membrane–binding matrix domain that reduced Gag-ESCRT binding increased Gag-plasma membrane binding and Gag release. ESCRT knockout mutants showed that the release enhancement was an ESCRT-dependent effect. Similarly, matrix mutation enhanced Gag release from human HEK293 cells. Release enhancement partly depended on ALIX binding to p6, although binding site mutation did not impair WT Gag release. Accordingly, the relative affinity for matrix compared with p6 in GST-pulldown experiments was higher for ALIX than for TSG101. We suggest that a transient matrix-ESCRT interaction is replaced when Gag binds to the plasma membrane. This step may activate ESCRT proteins and thereby coordinate ESCRT function with virion assembly.

HIV leaves the host cell by budding through the plasma membrane (PM) (reviewed in Ref. 1). The structural HIV polyprotein Gag drives this process. Gag-expressing cells release virus-like particles (VLPs), morphologically similar to immature HIV (2). Gag begins to oligomerize in the cytosol (3). Higher-order structures assemble at the PM, where Gag extends to its final rodlike conformation (3, 4, 5). The N terminus is located at the PM, and the C terminus is oriented toward the particle center (6). After budding, the viral protease cleaves Gag into the individual proteins, matrix (MA), capsid (CA), and nucleocapsid (NC), which then arrange into the mature virion (7). In Gag, they form separately folded domains, the structure and intermolecular contacts of which differ partly from those in the mature particle (8, 9, 10). The N-terminal MA directs Gag to the PM. MA consists of an N-terminal globular head and a C-terminal stalk (11). A basic amino acid (aa) cluster in the globular head makes specific contact with the PM by binding to phosphatidylinositol 4,5-bisphosphate (12, 13, 14, 15). A myristoyl chain that is enzymatically attached to glycine-2 after methionine-1 removal strengthens Gag-PM binding (7, 16, 17). In monomeric MA, the myristate moiety is sequestered in the globular head and is exposed upon MA trimerization and interaction with lipids (18, 19, 20). CA and NC are critical for oligomerization and immature lattice formation (21, 22).

The Gag C-terminal p6 peptide recruits ESCRT proteins (23, 24, 25, 26, 27). ESCRTs were first identified through genetic screens with the baker's yeast *S. cerevisiae* that investigated protein transport to the vacuole (28, 29, 30). In a process called the multivesicular body (MVB) pathway, the cytosolic ESCRT proteins bind to the endosomal membrane, capture substrates (often ubiquitinated membrane proteins), and induce budding and scission of substrate-containing vesicles into the endosomal lumen (31, 32). Thus, similar to HIV budding, they drive a budding process directed away from the cytosol.

The ESCRT machinery comprises four heterooligomeric core complexes (ESCRT-0 (Vsp27 and Hse1), ESCRT-I (Vps23, Vps28, Vps37, and Mvb12), ESCRT-II (Vps22, Vps25, and Vps36), and ESCRT-III (Snf7, Vps2, Vps20, and Vps24)), Bro1, the deubiquitinating enzyme Doa4, and the AAA-ATPase Vps4, which disassambles ESCRT complexes (32, 33, 34, 35, 36, 37, 38, 39, 40). Human cells express homologous proteins with several isoforms (27, 41). ESCRT-III is likely responsible for membrane remodeling (reviewed in Ref. 42). ESCRT-III proteins polymerize into spirals and tubes (43, 44, 45). Membrane-bound spirals are thought to deform the membrane by changing their diameters (45, 46, 47). Tubes could form an inside scaffold facilitating neck formation and scission (48). Whether Vps4 contributes to membrane remodeling or only recycles ESCRTs afterward is a matter of debate (49, 50, 51, 52, 53, 54). Two early acting factors, the ESCRT-I/II supercomplex and Bro1, nucleate ESCRT-III assembly, may stabilize membrane curvature via their protein surfaces, and recognize MVB substrates by binding to ubiquitin or specific peptide motifs (55, 56, 57, 58, 59, 60, 61, 62, 63, 64, 65, 66, 67, 68). ESCRT-0 also recognizes ubiquitinated cargo (37, 69). Its Vps27 subunit recruits ESCRT-I by binding to Vps23 and seems to have an additional ESCRT-I–independent function (68, 70, 71).

HIV budding and MVB-vesicle formation differ in that assembling Gag molecules form a curved lattice that may provide a scaffold for membrane deformation (72). Whether the ESCRTs are involved in generating membrane curvature in this process or only mediate viral membrane scission is controversial (23, 52, 73, 74). In any event, Gag assembly and ESCRT function must be coordinated to allow proper virion composition. Viral structural proteins recruit ESCRTs by common peptide motifs (reviewed in Ref. 75). A P(S/T)AP and a YP*X*_3_L motif in HIV-1 Gag p6 bind to the early acting proteins TSG101 and ALIX, the human Vps23 and Bro1 homologs (23, 24, 25, 26). Inasmuch as p6 is oriented toward the viral particle interior, it is unclear how ESCRTs recruited to p6 reach and manipulate the PM. Experiments using different methods indicate that ESCRT-I, -II, and -III, ALIX, and VPS4 are involved in HIV-1 release, although the results for some components are inconsistent (23, 26, 27, 76, 77, 78, 79, 80, 81). TSG101-binding site mutation strongly reduces virus release (23, 82). The role of the ALIX-p6 interaction is enigmatic. Binding site mutation in full-length Gag reduces HIV-1 release only weakly, whereas it strongly impairs the release when the MA globular head and the N-terminal CA domain (NCA) are deleted (26, 83, 84). A kinetic study shows that TSG101- and ALIX-binding site mutations in p6 only delay virus release, which indicates that ESCRTs are not absolutely necessary or may be recruited by additional sites (85).

We asked the question of whether yeast can be used to study the viral budding mechanism. Previous work showed that yeast spheroplasts release HIV-1 Gag VLPs (86, 87). However, VLP release was ESCRT-independent. We developed a different protocol and observed ESCRT-dependent HIV-1 Gag-GFP release from yeast spheroplasts. Binding assays with yeast ESCRT proteins revealed a previously unknown ESCRT interaction with the N-terminal Gag. We analyzed Gag-PM association and Gag release with Gag mutants and ESCRT knockout strains. Our results indicate a transient ESCRT-MA interaction that is replaced by Gag-PM binding. Our results further suggest that the MA interaction may block ESCRT function. This mechanism may contribute to spatiotemporally coordinate ESCRT action with Gag assembly. Based on our findings from yeast, we performed binding studies with human ESCRT proteins and Gag release assays with HEK293 cells. These studies confirmed our findings from yeast and help to clarify the enigmatic role of ALIX-p6 binding.

## Results

### Yeast Gag-GFP expression induces PM budding

**Figure 1:**
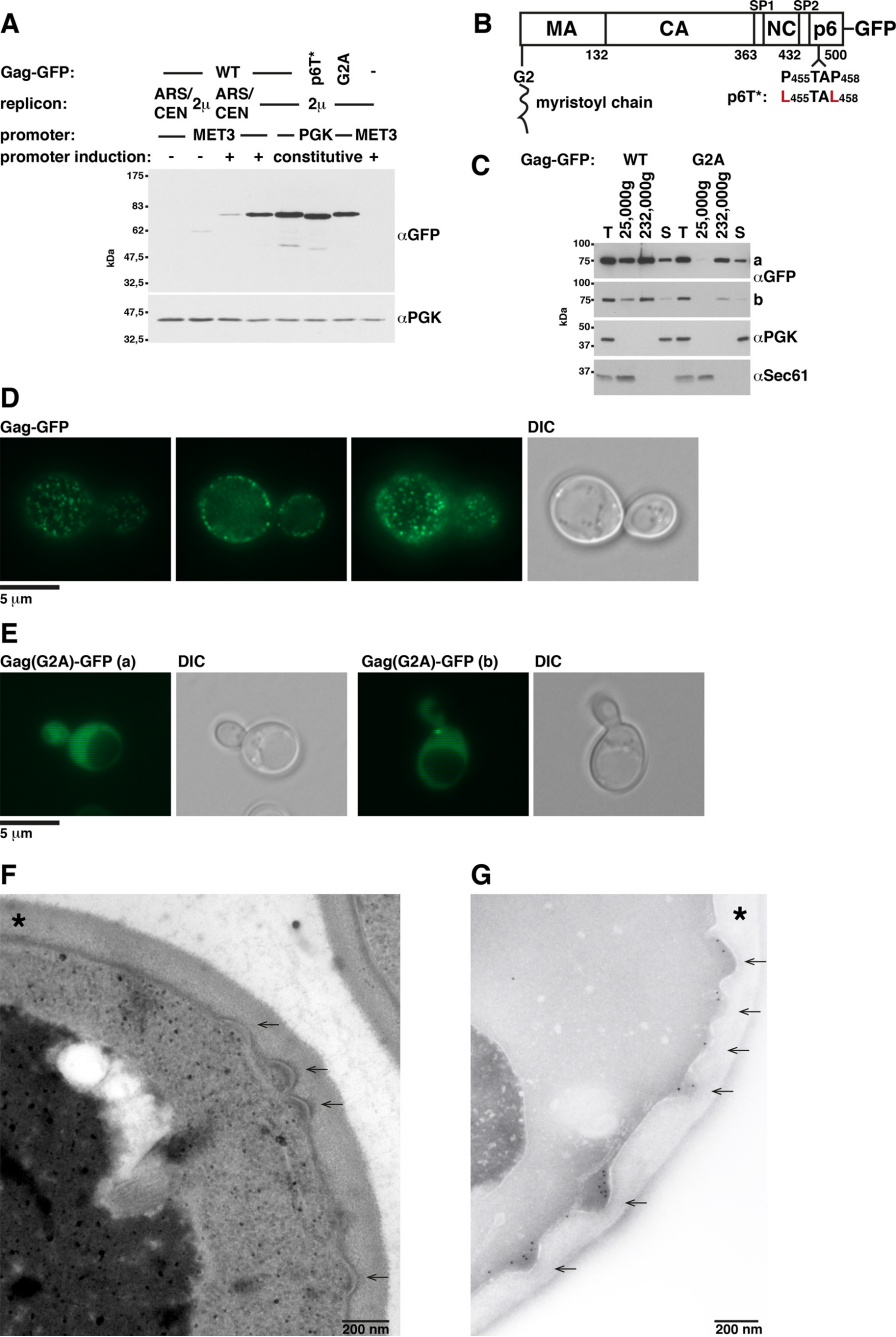
**HIV-1 Gag-GFP forms buds at the yeast PM.***A*, the Gag-GFP expression level was determined by cell extract immunoblotting. Gag-GFP, a version with mutated TSG101-binding site in p6 (p6T*), or a version with a mutated myristoylation site (G2A) was expressed in WT cells from a vector with constitutive PGK promoter and 2µ replicon or from a vector with MET3 promoter and 2µ or ARS/CEN replicon. Cells expressing MET3 promoter Gag-GFP were grown in medium containing 20 mg/liter methionine to repress the promoter. To induce the promoter, cells were shifted to medium lacking methionine for 4 h. PGK served as loading control. *B*, Gag-GFP schematic. *C*, Gag-GFP**–**membrane binding was analyzed by differential centrifugation, showing that the 25,000 × *g* pellet differentiates between PM-bound and cytosolic Gag. A 25,000 × *g* pellet, a 232,000 × *g* pellet, and a supernatant (*S*) were prepared from extracts (*T*) of WT cells expressing GFP-tagged Gag or Gag(G2A) from a 2µ vector with PGK promoter and analyzed by immunoblotting with the indicated antibodies. The pellets were concentrated (25,000 × *g*, 4×; 232,000 × *g*, 3.3×) compared with supernatant and extract samples. The cytosolic protein PGK and the integral ER membrane protein Sec61 served as references. A short (*b*) and a long (*a*) exposure are shown. *D* and *E*, the intracellular localization of GFP-tagged Gag or Gag(G2A) expressed from a 2µ vector with PGK promoter in WT cells was analyzed by fluorescence microscopy, showing that Gag accumulates in punctate structures at the PM dependent on its N-terminal myristoylation. Three layers of a yeast cell are shown for Gag. *DIC*, differential interference contrast. *F* and *G*, PM deformation (*arrows*) induced by Gag-GFP expression was analyzed by EM. *Asterisks*, cell wall. *F*, WT cells expressing Gag-GFP from a 2µ vector with MET3 promoter induced for 6 h were embedded in Epon. *G*, cryosections of WT cells (SUB62) expressing Gag-GFP from a 2µ vector with PGK promoter were prepared, and GFP was labeled with immunogold.

To identify the HIV-1 Gag expression level allowing VLP release from *S. cerevisiae*, we cloned the Gag-GFP coding sequence into various expression vectors. Gag-GFP transcription was driven by the constitutive phosphoglycerate kinase (PGK) promoter in a multicopy 2μ vector or by the inducible MET3 promoter, either in a 2μ or a low-copy number ARS/CEN vector. We verified Gag-GFP expression by immunoblotting using anti-GFP antibodies, showing the strongest signal for PGK promoter–driven expression (Fig. 1*A*). To analyze the intracellular Gag-GFP localization, we used fluorescence microscopy. High Gag-GFP expression from a 2μ vector (an example is shown for the PGK promoter–driven expression in Fig. 1*D* and Figs. S1 and S2) revealed punctate structures at the PM and a cytosolic fluorescence. Similar to human cells, Gag expressed in yeast gets modified with a myristoyl chain at its N-terminal glycine (88). This modification is required for Gag-PM association (89). When our WT expressed Gag(G2A)-GFP, a version lacking the myristoyl-acceptor glycine, we observed only the cytosolic fluorescence (Fig. 1*E* and Fig. S3). We additionally analyzed Gag-membrane binding by cell extract centrifugation, separating a membrane-containing sediment and a cytosol-containing supernatant (Fig. 1*C*). We could differentiate between membrane-associated Gag-GFP and Gag(G2A)-GFP with a sediment generated by centrifugation at 25,000 × *g*. A portion of both proteins sedimented at 232,000 × *g*. Cytosolic Gag oligomers or Gag that accumulated in undefined structures visible in a subpopulation of cells expressing Gag(G2A)-GFP (Fig. 1*Eb* and Fig. S3) may be the substrate of this sediment. To characterize the punctate Gag-GFP structures at the PM at a higher resolution, we analyzed Epon-prepared yeast sections by EM (Fig. 1*F* and Figs. S4 and S5). Gag-GFP expression induced buds at the PM, whereas we did not observe these structures in cells carrying the empty vector. To clarify whether these buds contain the accumulating Gag-GFP, we performed cryosections and detected GFP by immunogold labeling, thereby exhibiting Gag-GFP inside the buds (Fig. 1*G* and Fig. S6). This approach does not exclude the recognition of potential GFP-containing breakdown products of Gag-GFP.

### ESCRTs determine Gag release from yeast spheroplasts

**Figure 2:**
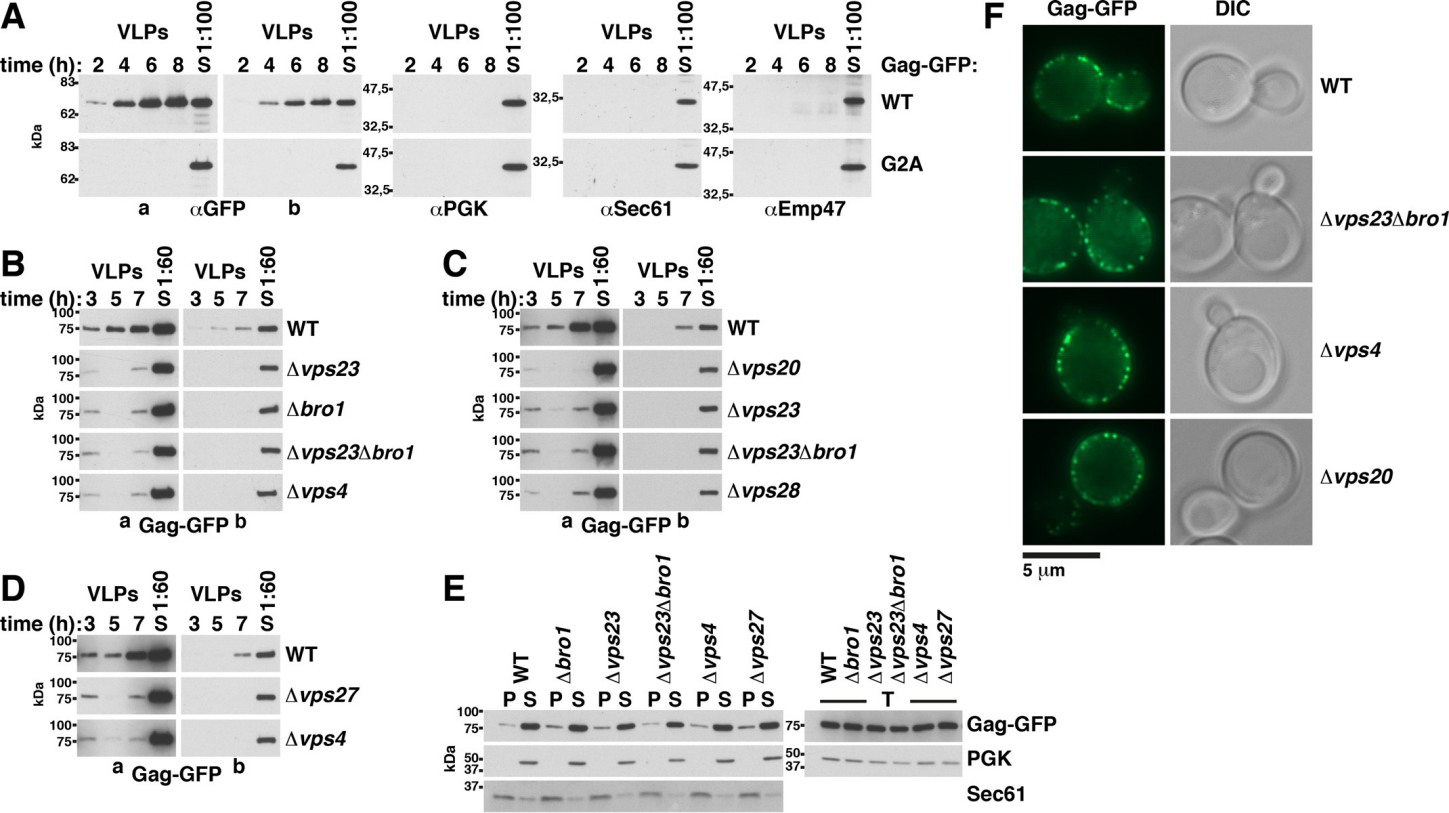
**Gag-GFP release from yeast spheroplasts depends on ESCRT proteins.***A*, Gag-GFP or Gag(G2A)-GFP release from WT yeast spheroplasts was analyzed by immunoblotting of high-speed centrifugation sediments derived from the incubation medium with anti-GFP antibodies (VLPs). VLPs were harvested every second hour after spheroplast preparation over a period of 8 h. Spheroplasts were incubated in fresh medium after each harvest. Release increased from the first to the third time point and required Gag myristoylation. Immunoblots with antibodies detecting PGK (cytosolic protein), Sec61 (ER membrane protein), or Emp47 (Golgi and COPII membrane protein) served as control for specific Gag release. Long (*a*) and short (*b*) exposures are shown. *S*, lysate of spheroplasts prepared at the final VLP harvest. *B–D*, same as *A* except that Gag-GFP was expressed in the WT or the indicated mutants and VLPs were harvested at the indicated times, showing ESCRT-dependent release. *E* and *F*, ESCRT deletion does not affect Gag-GFP-PM accumulation. *E*, Gag-GFP**-**membrane binding was analyzed by immunoblotting of membrane-containing 25,000 × *g* pellets (*P*) and cytosol-containing supernatants (*S*) derived from cell extracts (*T*). Sec61 and PGK served as references. *F*, Gag-GFP accumulation in punctate structures at the PM was analyzed by fluorescence microscopy. *DIC*, differential interference contrast. *A–F*, WT cells or the indicated mutants expressed Gag-GFP or Gag(G2A)-GFP from a 2µ vector with PGK promoter.

**Figure 4:**
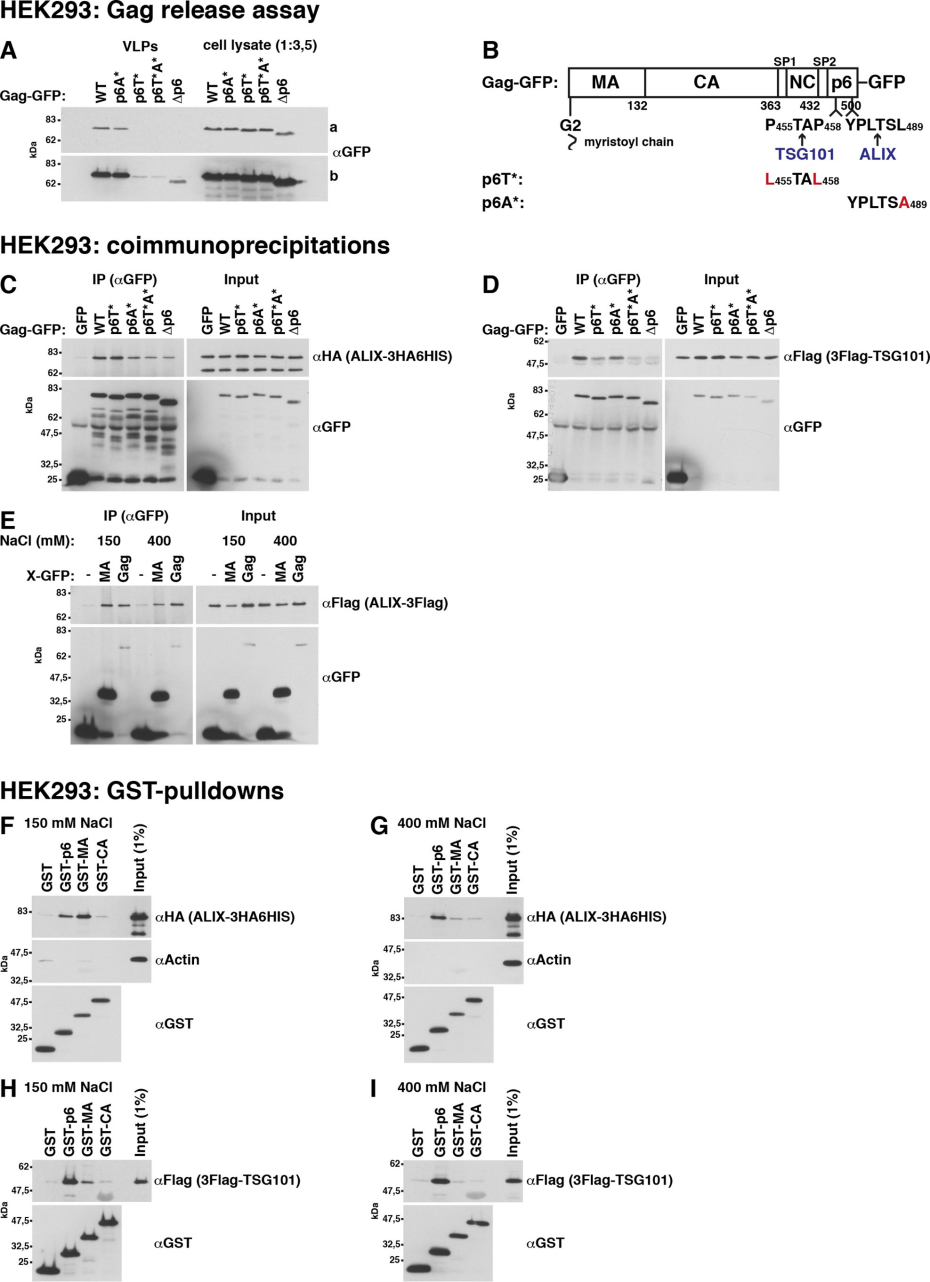
**ALIX and TSG101 bind to MA. The relative affinity for MA compared with p6 is higher for ALIX.***A*, Gag-GFP-VLP release from HEK293 cells recapitulating published data for p6 dependence of HIV-1 release. 2 days after transfection with CMV promoter expression vectors for the indicated Gag versions, VLPs were harvested from the culture medium, and cell lysates were prepared. Gag was detected by immunoblotting with anti-GFP antibodies. A short (*a*) and a long (*b*) exposure are shown. *B*, Gag-GFP schematic. *C–E*, coimmunoprecipitations (*IP*) of epitope-tagged ALIX or TSG101 with GFP-tagged Gag versions or MA expressed from CMV promoter vectors in HEK293 cells, indicating that ALIX binding to Gag is reduced but not prevented by p6 deletion and that ALIX binds to MA. TSG101 binding to Gag more strongly depends on p6. GFP-tagged proteins were immunoprecipitated with anti-GFP antibodies in the presence of 150 mm NaCl (*C* and *D*) or as indicated (*E*), and coimmunopreciptated ALIX or TSG101 was detected with antibodies recognizing the epitope tag. *F–I*, pulldown experiments showing that ALIX and TSG101 bind to GST-MA expressed in *E. coli*. The relative affinity for MA compared with p6 is higher for ALIX. GST-tagged Gag fragments (MA (aa 1–132), CA (aa 133–363), and p6 (aa 448–500)) were bound to GSH-Sepharose and incubated with extract of HEK293 cells expressing epitope-tagged ALIX or TSG101 from a CMV promoter vector. Bead-bound proteins were analyzed by immunoblotting with the indicated antibodies. α-Actin served as control, showing specific ESCRT protein binding.

In contrast to human cells, yeast cells are enclosed by a cell wall, which would prevent VLP release (Fig. 1, *F* and *G*). To test whether yeast can generate Gag VLPs after cell wall removal, we prepared yeast spheroplasts by enzymatic cell wall digestion and incubated the spheroplasts in medium containing 1 m sorbitol for osmotic stabilization. To harvest VLPs, we filtrated the medium through 0.45-μm pores to eliminate cell debris and collected VLPs by high-speed centrifugation. We used the same method to harvest Gag-GFP released from HEK293 cells (see Fig. 4*A*). In the latter experiment, the amount of collected Gag-GFP strongly depended on p6, consistent with HIV-1 release from human cells (82). Our result indicates that this method is valid to harvest VLPs. We prepared yeast spheroplasts that highly expressed Gag-GFP from a 2μ vector with PGK promoter (Fig. 2*A*). We harvested VLPs every second hour for a period of 8 h and incubated the spheroplasts in fresh medium after each harvest. Gag-GFP release strongly increased from the first to the third time point. We did not detect cellular proteins, the cytosolic PGK and two membrane proteins (the ER protein Sec61 and the Golgi protein Emp47), in the VLP sediments. This finding indicates that Gag-GFP was specifically released. We could not harvest VLPs when spheroplasts expressed Gag(G2A)-GFP, suggesting that Gag-GFP release depended on its prior binding to the PM. The results were similar when spheroplasts expressed Gag-GFP and Gag(G2A)-GFP from the 2μ vector with inducible MET3 promoter and when promoter induction started upon spheroplast preparation (Fig. S7C). We could not harvest VLPs when spheroplasts weakly expressed Gag-GFP from an ARS/CEN vector (Fig. S7E).

To test whether Gag-GFP release from yeast depends on ESCRTs, we analyzed knockout mutants of VPS27 (ESCRT-0), VPS23 and VPS28 (both ESCRT-I), VPS20 (ESCRT-III), BRO1, and VPS4 (Fig. 2 (*B–D*) and Fig. S7 (D and F–H)). During the first 3 h after spheroplast preparation, ESCRT mutants released a similar or modestly reduced Gag-GFP amount compared with WT, whereas the amount was strongly diminished during the following 4 h. These results suggest that the initial weak Gag-GFP release was partly ESCRT-independent and that the following strong release required the ESCRTs. To test whether impaired Gag assembly might be the reason for reduced VLP release from ESCRT mutants, we analyzed Gag-membrane binding by preparing a membrane-containing sediment and used fluorescence microscopy (Fig. 2, *E* and *F*). The Gag-GFP amount sedimenting with membranes was not reduced in ESCRT mutants. We observed the same pattern of punctate structures at the PM in ESCRT mutants as in WT cells. Moreover, the Gag-GFP membrane association kinetic after MET3 promoter induction was unaffected in ESCRT mutants (Fig. S7, A and B). Similar Gag-GFP amounts were in the membrane sediment prepared within the first 3 h after MET3 promoter induction. Thus, our results indicate that efficient Gag-GFP release from yeast requires ESCRTs, whereas Gag assembly does not. This finding is consistent with the accumulation of release-arrested, assembled HIV-1 virions in human cells with impaired ESCRT machinery and no microscopic detectable delay of Gag assembly at the human PM when the ESCRT-binding sites in p6 are deleted (23, 27, 90).

### Gag binds to yeast ESCRT proteins

**Figure 3:**
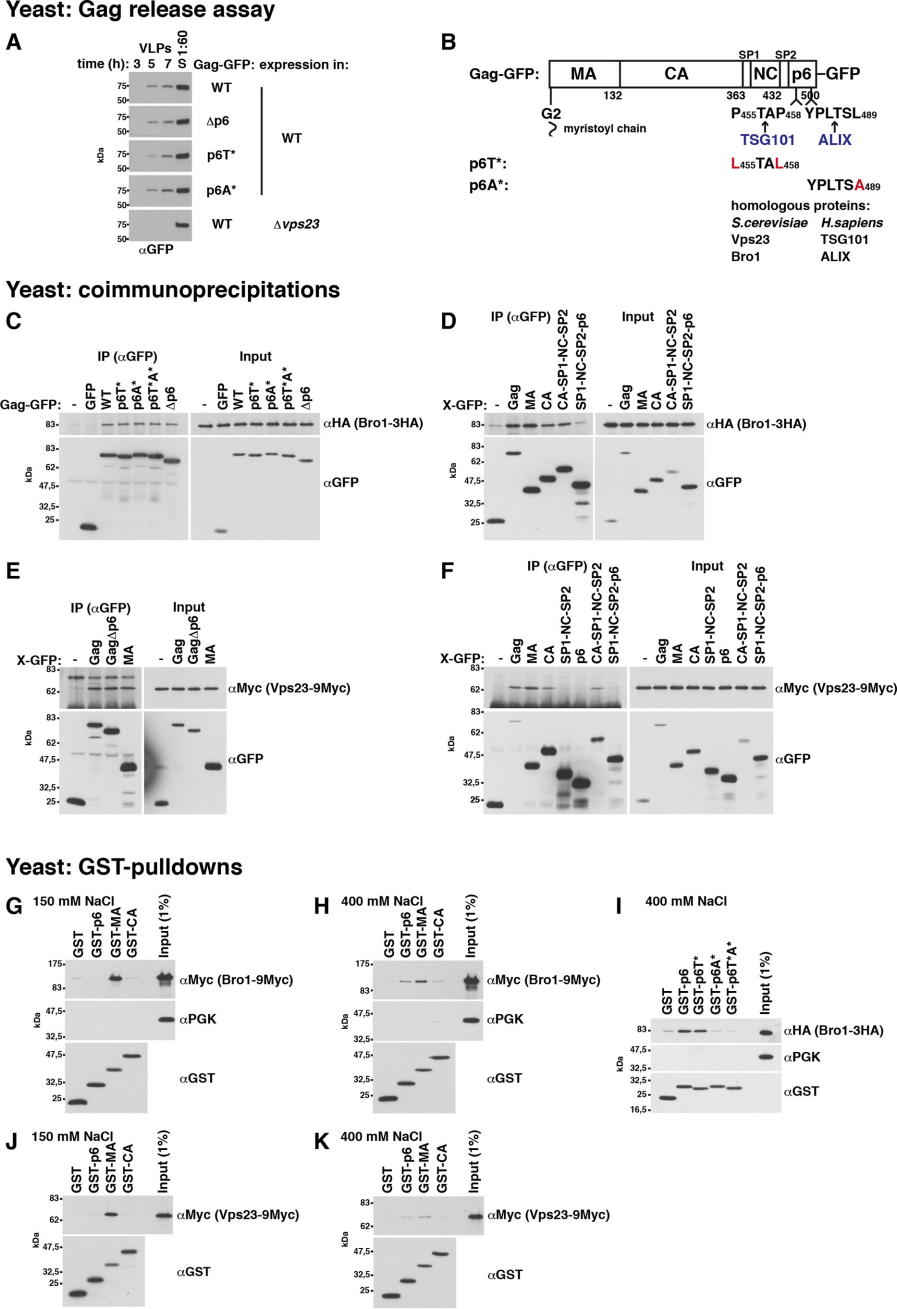
**Yeast ESCRT proteins bind to the Gag N-terminal protein region.***A*, release of Gag-GFP or the indicated mutants from WT yeast spheroplasts or a Δ*vps23* mutant was analyzed by immunoblotting of high-speed centrifugation sediments derived from the incubation medium with anti-GFP antibodies (VLPs), showing that p6 is not required for Gag-GFP release from yeast. Gag-GFP versions were expressed from a 2µ vector with PGK promoter. *S*, lysate of spheroplasts prepared at the final VLP harvest. *B*, Gag-GFP schematic. *C–F*, coimmunoprecipitation (*IP*) experiments with GFP-tagged Gag versions or Gag fragments expressed from a 2µ vector with induced MET3 promoter and genomically epitope-tagged Bro1 or Vps23, indicating that Bro1 and Vps23 bind to Gag via MA and CA. Gag versions or Gag fragments (MA (aa 1–132), CA (aa 133–363), p6 (aa 448–500), CA-SP1-NC-SP2 (aa 133–447), SP1-NC-SP2 (aa 364–447), SP1-NC-SP2-p6 (aa 364–500), and GagΔp6 (aa 1–447)) were immunoprecipitated with anti-GFP antibodies in the presence of 400 mm NaCl, and coimmunoprecipitated Bro1 or Vps23 was detected by immunoblotting with anti-HA or anti-Myc antibodies. *C*, Bro1 binds to Gag independent of p6. *D*, Bro1 binds to Gag fragments containing MA or CA. *E*, Vps23 binds to Gag independent of p6 and to MA. *F*, Vps23 binds to Gag fragments containing MA or CA. *G–K*, pulldown experiments showing that genomically epitope-tagged Bro1 and Vps23 bind to GST-MA expressed in *E. coli* and that Bro1 additionally binds to GST-p6 dependent on the ALIX-binding motif. GST-tagged Gag fragments (MA (aa 1–132), CA (aa 133–363), and p6 (aa 448–500)) were bound to GSH-Sepharose and incubated with yeast extract. Bead-bound proteins were analyzed by immunoblotting with the indicated antibodies. PGK served as control, showing specific ESCRT protein binding. *G* and *J*, binding buffer contained 150 mm NaCl. *H*, *I*, and *K*, binding buffer contained 400 mm NaCl.

The human ESCRT proteins ALIX and TSG101 bind to p6 via motifs resembling cellular substrate or interaction partner sequences (66, 91, 92, 93, 94). A mutated TSG101-binding site in p6 strongly reduces HIV-1 release, whereas the ALIX-binding site is less important (23, 82, 83, 84). Our Gag-GFP release assay with HEK293 cells recapitulated these results (see Fig. 4*A*). In addition, p6 deletion or mutation of the specific binding site in p6 (p6T* (24), p6A* (26)) strongly reduced the CMV promoter–expressed TSG101 or ALIX amount that coimmunoprecipitated with Gag-GFP (Fig. 4, *C* and *D*). The yeast TSG101 and ALIX homologs, Vps23 and Bro1, recognize similar but not identical motifs in yeast proteins (70, 95, 96). Therefore, we asked whether yeast ESCRTs physically interact with Gag. We could pull down Bro1 from a yeast extract with GST-p6 expressed in *Escherichia coli*, but not with GST-p6A* (Fig. 3*I*). This finding indicates that Bro1 binds to the same site in p6 as its human homolog. In addition, Bro1 coimmunoprecipitated with Gag-GFP expressed in yeast (Fig. 3*C*). Unexpectedly, this interaction was independent of p6. Similarly, the same Vps23 amounts coimmunoprecipitated with Gag(Δp6)-GFP and Gag-GFP (Fig. 3*E*). In contrast to Bro1, we could not pull down Vps23 with GST-p6 (Fig. 3, *K versus H*). Thus, our results indicate that yeast ESCRT proteins bind with higher affinity to a protein region outside of p6. These results are consistent with the finding that Gag-GFP release from yeast, while ESCRT-dependent, did not require p6 (Fig. 3*A*).

### Yeast and human ESCRT proteins bind to the Gag N-terminal protein region

To identify the Gag domain that interacts with yeast ESCRT proteins, we immunoprecipitated GFP-tagged Gag fragments and tested for coimmunoprecipitating Bro1 and Vps23 (Fig. 3 (*D* and *F*) and Fig. S8A). Both proteins coimmunoprecipitated with Gag fragments containing either MA or CA. Moreover, we could pull down Bro1 and Vps23 with GST-MA (Fig. 3, *G* and *J*). This finding indicates that the ESCRT-MA interaction does not require a posttranslational MA modification that does not occur in *E. coli*, namely ubiquitination. Bro1 and Vps23 did not bind to GST-CA. This may indicate that the interaction with CA requires a CA posttranslational modification or may be simply due to structural differences between GST-CA and CA-GFP. Human ESCRT binding to the Gag N-terminal protein region has not been described. We could pull down ALIX and TSG101 with GST-MA from an extract of HEK293 cells expressing these ESCRT proteins via a CMV promoter (Fig. 4 (*F* and *H*) and Fig. S9 (G–J)). Less TSG101 bound to GST-MA than to GST-p6, whereas a similar ALIX amount bound to GST-MA compared with GST-p6. Moreover, we coimmunoprecipitated ALIX with MA-GFP expressed in HEK293 cells (Fig. 4*E*). Although the ALIX amount that coimmunoprecipitated with Gag-GFP depended on p6, a significant amount still bound to Gag(Δp6)-GFP (Fig. 4*C*). This finding additionally indicates that ALIX can bind to a site other than p6.

As we observed salt-dependent binding in our GST-pulldown experiments, we performed experiments in the presence of 150 and 400 mm NaCl. Binding of Bro1, Vps23, and their human homologs to GST-MA was strongly reduced at 400 mm NaCl (Figs. 3 (*G*, *H*, *J*, and *K*) and 4 (*F–I*)). In contrast, we could pull down Bro1 with GST-p6 only in presence of 400 mm NaCl (Fig. 3, *H versus G*), whereas both human ESCRT proteins, ALIX and TSG101, bound to p6 in the presence of 150 and 400 mm NaCl (Fig. 4, *F–I*). Inasmuch as yeast ESCRT proteins bound to MA-GFP in the presence of 400 mm NaCl and we did not want to prevent a potential ESCRT binding to Gag-GFP via p6, we performed our subsequent yeast coimmunoprecipitation experiments with incubation buffer containing 400 mm NaCl. For coimmunoprecipitation experiments with Gag-GFP expressed in HEK293 cells, results were similar in the presence of 150 and 400 mm NaCl (Fig. 4*C versus* Fig. S8C and Fig. 4*D versus* Fig. S8D).

RNA-dependent ALIX binding to the Gag NC domain was described (97). We did not observe NC-specific Bro1 binding to Gag fragments (Fig. 3*D* and Fig. S8A). Because Vps23 coimmunoprecipitated with an NC-containing Gag fragment at 150 mm NaCl (Fig. S8B), we cannot exclude the possibility that a Bro1-NC interaction might occur under salt conditions lower than the 400 mm NaCl used in our assays.

The results show that Bro1, Vps23, and their human homologs bind to the Gag N-terminal protein region. Bro1 and Vps23 bound to MA and CA in coimmunoprecipitation experiments. We confirmed binding to MA with a GST-pulldown experiment. ALIX coimmunoprecipitated with MA-GFP and could be pulled down with GST-MA. TSG101 bound with lower affinity to GST-MA compared with GST-p6.

### MA hydrophobic-patch mutations reduce MA binding to yeast ESCRT proteins

**Figure 7:**
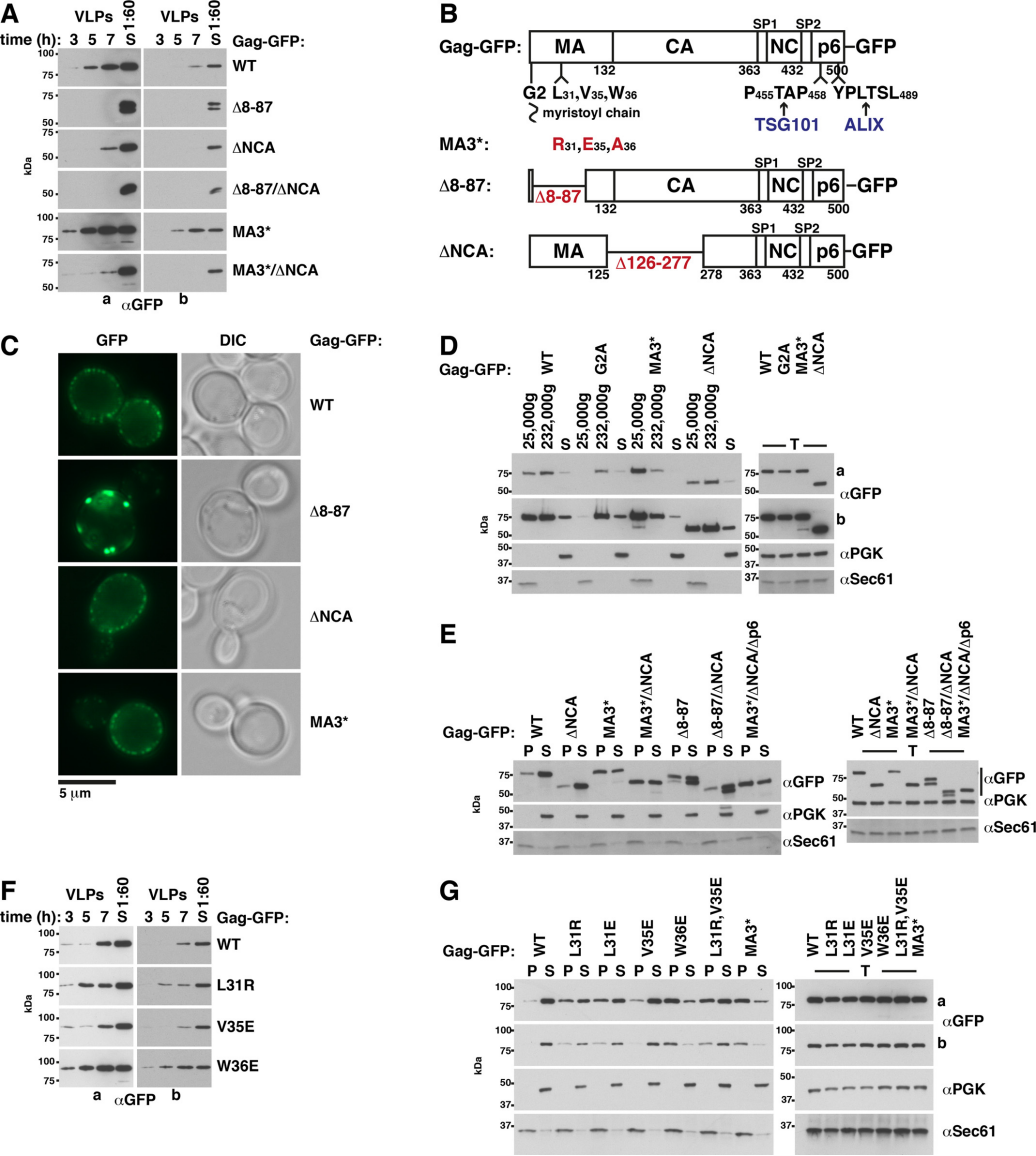
An MA hydrophobic patch mutation (MA3*) increases Gag-GFP-membrane binding and enhances Gag-GFP release from yeast.*A*, release of Gag-GFP versions from yeast spheroplasts was analyzed by immunoblotting of high-speed centrifugation sediments derived from the incubation medium with anti-GFP antibodies (VLPs), showing that MA3* increases Gag release. ΔNCA reduces Gag release and is dominant over MA3*. MA globular head deletion (Δ8–87) abrogates Gag release. *S*, lysate of spheroplasts prepared at the final VLP harvest. Long (*a*) and short (*b*) exposures are shown. *B*, Gag-GFP schematic. *C*, PM accumulation of Gag-GFP versions was analyzed by fluorescence microscopy, showing that Gag(MA3*) and Gag(ΔNCA) form punctate structures similar to Gag, whereas Gag(Δ8-87) forms PM-associated aggregates with larger diameter. *DIC*, differential interference contrast. *D*, membrane binding of Gag-GFP versions was analyzed by differential centrifugation, showing that MA3* increases specifically the Gag amount that sediments with the 25,000 × *g* membrane pellet. Gag(ΔNCA) sediments similarly to Gag. A 25,000 × *g* pellet, a 232,000 × *g* pellet, and a supernatant (*S*) were prepared from cell extracts (*T*) and analyzed by immunoblotting with the indicated antibodies. The pellets were concentrated (25,000 × *g*, 4×; 232,000 × *g*, 3.3×) compared with supernatant and extract samples. The cytosolic protein PGK and the integral ER membrane protein Sec61 served as references. A short (*a*) and long (*b*) exposure are shown. *E*, membrane-containing 25,000 × *g* pellets (*P*) and cytosol-containing supernatants (*S*) derived from cell extracts (*T*) were analyzed as in *D*, showing that increased amounts of Gag versions carrying the MA3* mutation sediment with membranes compared with WT Gag, whereas the ΔNCA mutation does not affect the membrane association. *G*, same as *E*, except that cells expressed the indicated Gag-GFP versions, showing that Leu-31 and Trp-36 mutations increase Gag-membrane binding. A long (*a*) and a short (*b*) exposure are shown. *F*, same as *A*, except that the indicated Gag-GFP versions were expressed, showing that Leu-31 and Trp-36 mutations increase Gag release. *A* and *C–G*, Gag-GFP versions were expressed from a 2µ vector with PGK promoter in WT yeast.

**Figure 9:**
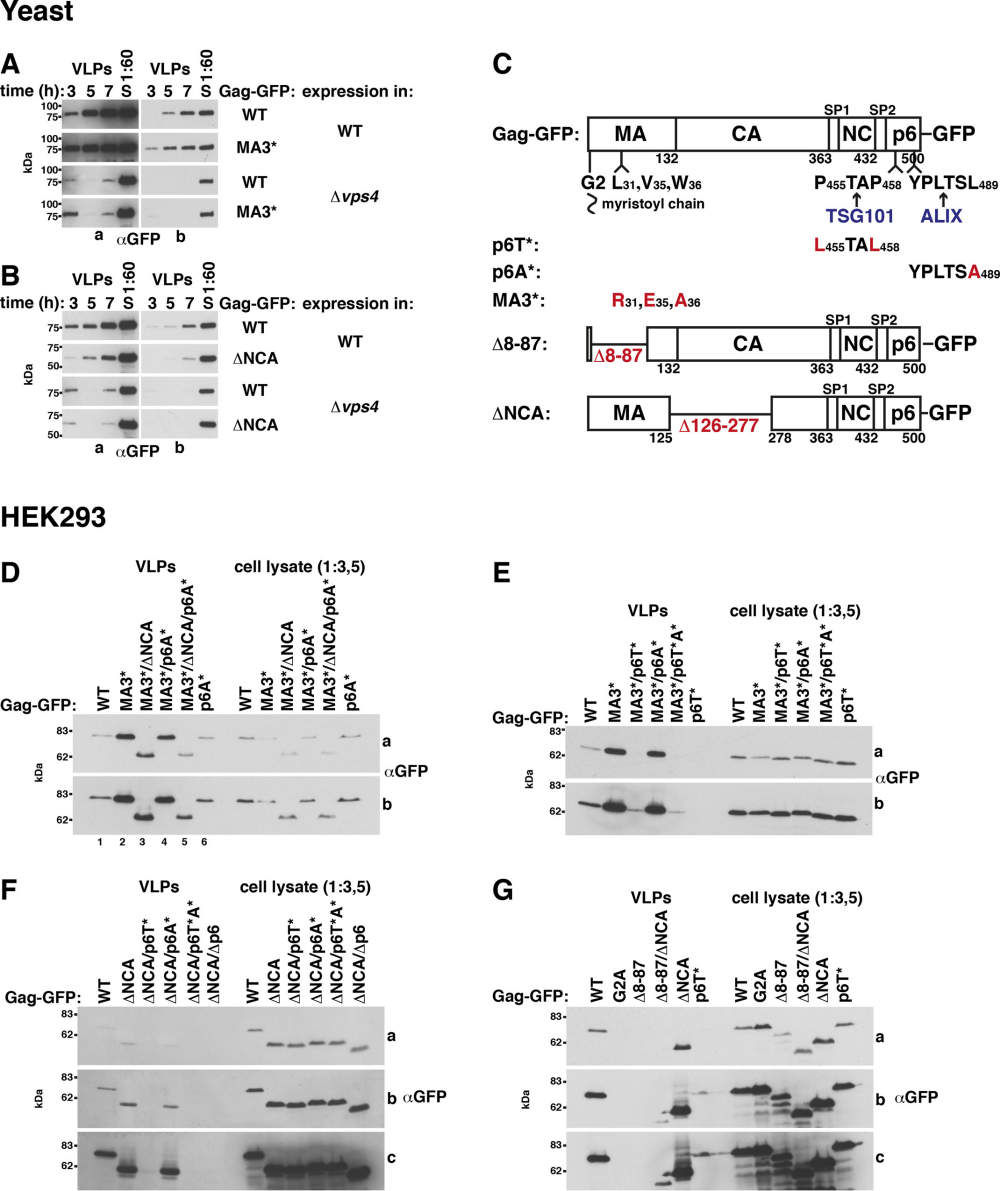
Increased Gag(MA3*)-GFP release from yeast depends on ESCRT proteins; MA3* increases Gag-GFP release from HEK293 cells dependent on NCA and the ALIX-binding site in p6.*A* and *B*, release of Gag-GFP versions from yeast spheroplasts was analyzed by immunoblotting of high-speed centrifugation sediments derived from the incubation medium with anti-GFP antibodies (VLPs). Gag-GFP versions were expressed from a 2µ vector with PGK promoter. *S*, lysate of spheroplasts prepared at the final VLP harvest. Long (*a*) and short (*b*) exposures are shown. *A*, VPS4 deletion abrogates the release-increasing MA3* effect after 5 and 7 h of incubation. *B*, Gag(ΔNCA)-GFP release from Δ*vps4* spheroplasts is slightly decreased compared with Gag-GFP. *D–G*, Gag-GFP release from HEK293 cells 2 days after transfection with CMV promoter expression vectors for the indicated Gag versions. VLPs were harvested from the culture medium, and cell lysates were prepared. Gag-GFP was detected by immunoblotting with anti-GFP antibodies. Several exposures (*a–c*) are shown. *D*, the MA3* mutation increases Gag-GFP release. A combination of ΔNCA and mutation of the ALIX-binding site in p6 (p6A*) abrogates this increase, whereas an isolated p6A* mutation does not impair Gag-GFP release. *E*, similar to Gag-GFP, mutating the TSG101-binding site in p6 (p6T*) strongly reduces Gag(MA3*)-GFP release. *F* and *G*, Gag(ΔNCA) is efficiently released. *F*, similar to Gag-GFP, p6T* strongly reduces Gag(ΔNCA)-GFP release. *G*, MA globular head (Δ8–87) deletion strongly impairs the release. *C*, Gag-GFP schematic.

Because our binding studies indicated that MA interacts with yeast and human ESCRT proteins, we searched for MA mutants that reduced the MA-ESCRT interaction to analyze their impact on VLP release. Published data suggest that Gag(Δ8-87), a mutant lacking most of the MA globular head, does not impair virion release (98). In contrast, we could not harvest Gag(Δ8-87)-GFP VLPs from yeast spheroplasts, and aa 8–87 deletion strongly reduced Gag-GFP release from HEK293 cells (see Figs. 7*A* and 9*G*). Gag(Δ8-87)-GFP accumulated at the yeast PM in structures with larger diameters and lower number than WT Gag-GFP (Fig. 7*C* and Fig. S10). In HEK293 cells, Gag(Δ8-87)-GFP formed many small accumulations, not visible for Gag-GFP (not shown). The presence of an additional band of unknown nature in the Gag(Δ8-87)-GFP immunoblot might explain the assembly defect (Fig. 7, *A* and *E*). The findings show that Gag(Δ8-87)-GFP was unsuitable for our experiments.

**Figure 5:**
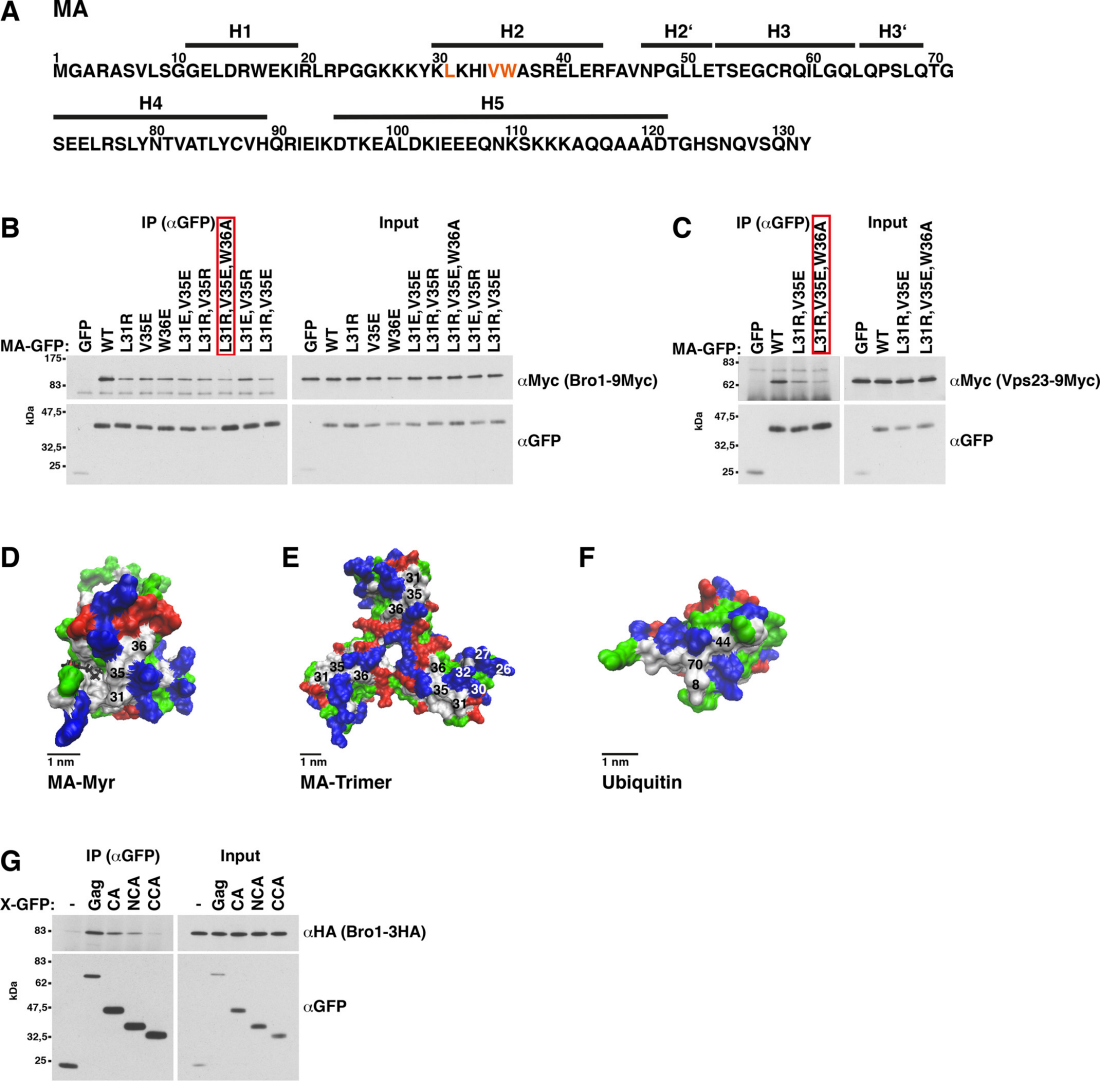
**Mutation of an MA hydrophobic patch consisting of Leu-31, Val-35, and Trp-36 reduces Bro1 and Vps23 binding to MA; Bro1 binds via NCA to CA.***A*, MA protein sequence derived from pGag-EGFP (130) used as template in this study. Leu-31, Val-35, and Trp-36, the mutation of which reduced binding to Bro1, are marked in *orange*. Helix (*H*) assignments are derived from the MA X-ray structure (11). *B* and *C*, GFP-tagged MA versions were expressed from a 2µ vector with induced MET3 promoter in yeast cells carrying genomically 9Myc-tagged Bro1 or Vps23. MA was immunoprecipitated (*IP*) in the presence of 400 mm NaCl with anti-GFP antibodies, and coimmunoprecipitated Bro1 or Vps23 was detected by immunoblotting with anti-Myc antibodies. The *red box* indicates mutant MA3* that was chosen in subsequent experiments to diminish the ESCRT-MA interaction. *B*, Leu-31, Val-35, or Trp-36 mutation reduces the MA-Bro1 interaction. *C*, MA mutants that reduce the binding to Bro1 also diminish the MA-Vps23 interaction. *D–F*, molecular surface structures visualized with the VMD software. *White*, nonpolar aa; *blue*, basic aa; *red*, acidic aa; *green*, polar aa; *black*, myristoyl residue. *D*, MA NMR structure (PDB entry 2H3I) (19), showing that Leu-31, Val-35, and Trp-36 form a hydrophobic patch on the MA surface. *E*, MA trimer X-ray structure (PDB entry 1HIW) (11), showing that Leu-31, Val-35, and Trp-36 are located on the MA side that is exposed to the PM and are not part of the trimerization interface. Basic aa 26, 27, 30, and 32 were proposed to be involved in MA binding to phospholipids (13, 99, 101). *F*, human ubiquitin X-ray structure (PDB entry 1UBQ) (135), showing that the hydrophobic patch in MA consisting of Leu-31, Val-35, and Trp-36 resembles the hydrophobic ubiquitin patch consisting of Leu-8, Ile-44, and Val-70 which is involved in ubiquitin binding to Vps23, Bro1, TSG101, and ALIX (58, 67, 102). *G*, Bro1 coimmunoprecipitation with GFP-tagged CA versions, showing that Bro1 binds to the N-terminal CA domain; same as *B* except that GFP-tagged CA, NCA (aa 133–278), or CCA (aa 279–363) was expressed in a yeast strain carrying genomically 3HA-tagged Bro1 and that coimmunoprecipitated Bro1 was detected by immunoblotting with anti-HA antibodies.

We then searched MA point mutations that reduced the interaction with Bro1. We coimmunoprecipitated Bro1 with MA-GFP versions expressed in yeast. We began with MA subfragments and internal-deletion mutants (examples in Fig. S11 (A–C)). The results indicated that helix-2 and the strand loop between helix-1 and -2 could be involved in the MA-Bro1 interaction (Fig. 5*A*). Because the three-dimensional structure of the tested MA versions might be impaired, we additionally performed an AAA-scanning mutagenesis for residues 20–43 (Fig. S11, D–F). The expression levels of AAA mutants spanning aa 23–43 were similar to WT MA-GFP. Two mutants, YKL29,30,31AAA and VWA35,36,37AAA, reduced the MA-Bro1 interaction. Next, we prepared single exchanges of aa 29–31 and 35–37. In addition to Ala, we tested exchanges against Arg, Glu, or Trp. Leu-31, Val-35, or Trp-36 mutations diminished the MA-Bro1 binding (Fig. 5*B*). Based on the MA NMR and X-ray structures and lipid-interaction studies (11, 13, 19, 99, 100, 101), these three nonpolar aa are exposed on the MA surface, are not part of the MA trimerization interface, and are located on the globular head side that faces the PM (Fig. 5, *D* and *E*). To further diminish the interaction, we combined mutations. For the subsequent experiments, we chose MA3* (L31R,V35E,W36A), a mutant in which we exchanged the 3 uncharged aa against a basic, an acidic, and a neutral aa, to keep the change of the overall molecule charge small. Our results do not exclude the possibility that other MA sites may contribute to the interaction.

In addition to Bro1, MA3* reduced the MA-Vps23 binding (Fig. 5*C*). Because we coimmunoprecipitated or pulled down the ESCRT proteins in our binding studies from cell extract, we cannot exclude the possibility that MA recruited Vps23 or Bro1 by another factor, rather than binding directly. The following options exist: first, either Bro1 or Vps23 bound directly to MA and recruited the other protein; second, MA recruited both ESCRT proteins by another factor; or third, Bro1 and Vps23 bound directly to the same site in MA. To test whether Vps23 binds via Bro1 to MA or vice versa, we performed pulldown experiments with GST-MA and extract of Δ*bro1* or Δ*vps23* cells (Fig. S12, D and E). The Vps23-MA interaction was independent of Bro1, and vice versa. We obtained similar results for coimmunoprecipitation experiments with Gag-GFP and MA-GFP (Fig. S12, A–C). These results indicate that Bro1 and Vps23 bound either both directly to MA or were recruited by another factor. The three-dimensional structure of the hydrophobic patch formed by Leu^31^, Val^35^, and Trp^36^ resembles the ubiquitin hydrophobic patch that mediates the ubiquitin interaction with Vps23, Bro1, and their human homologs (Fig. 5*F*) (58, 67, 102). This fact may explain the direct binding of both ESCRT proteins, Bro1 and Vps23, to MA.

### Mutations in the Gag N-terminal protein region reduce yeast ESCRT protein binding

**Figure 6:**
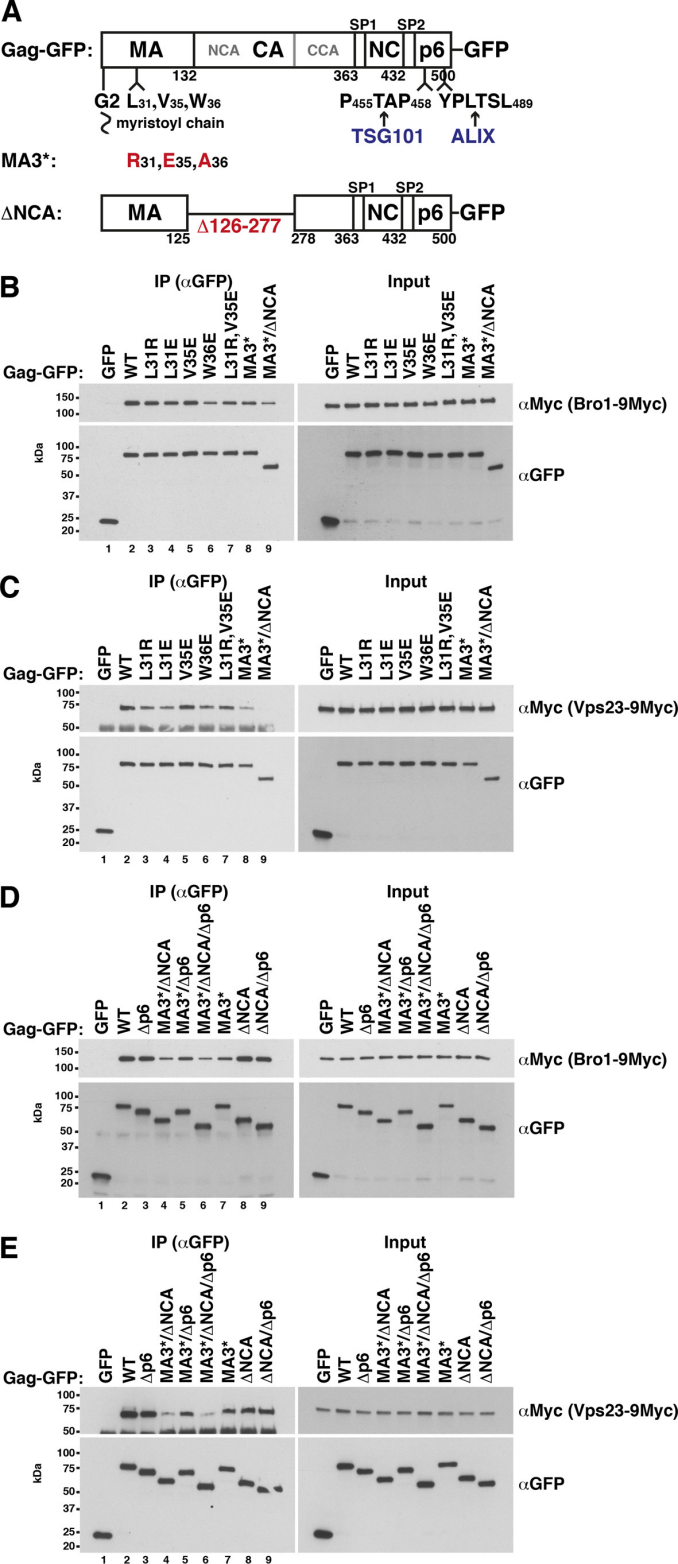
**Mutations in the Gag N-terminal protein region reduce Gag-Bro1 and Gag-Vps23 interaction.***B–E*, coimmunoprecipitation (*IP*) experiments with Gag-GFP versions expressed from a 2µ vector with induced MET3 promoter in yeast cells carrying genomically 9Myc-tagged Bro1 or Vps23, showing that MA mutations in Leu-31 and Trp-36 slightly reduce the Gag-ESCRT binding. aa126-277 deletion (ΔNCA) enhances this effect (Gag(MA3*)-GFP versus Gag(MA3*/ΔNCA-GFP). V35E does not reduce the Gag-ESCRT coimmunoprecipitation. Gag was immunoprecipitated with anti-GFP antibodies in the presence of 400 mm NaCl, and coimmunoprecipitated Bro1 or Vps23 was detected by immunoblotting with anti-Myc antibodies. *A*, Gag-GFP schematic.

Next, we introduced Leu-31, Val-35, and Trp-36 mutations into Gag-GFP. With the exception of V35E, all mutations slightly reduced the Bro1 and Vps23 amounts that coimmunoprecipitated with Gag-GFP (Fig. 6 (*B* and *C*) and Fig. S13J). Because our binding studies with isolated Gag domains suggested that yeast ESCRT proteins can bind to MA, CA, and p6 (Fig. 3), we asked whether preventing ESCRT interaction with CA and p6 would further decrease ESCRT binding to Gag(MA3*)-GFP. CA consists of two independently folded domains (10). The C-terminal domain (CCA) is crucial for Gag assembly, whereas deleting aa 126–277 (Gag(ΔNCA)), which comprise NCA and the 7 C-terminal MA stalk aa, does not impair HIV-1 release (21, 103). This situation is consistent with efficient Gag(ΔNCA)-GFP release from HEK293 cells in our experiments (see Fig. 9, *F* and *G*) and indicates that deleting aa 126–277 does not significantly disturb Gag assembly. Inasmuch as Bro1 coimmunoprecipitated with NCA and not with CCA (Fig. 5*G*), we deleted aa 126–277 to abrogate yeast ESCRT binding to CA in our subsequent experiments. ΔNCA reduced ESCRT binding to Gag(MA3*)-GFP (Fig. 6 (*B* and *C* (*lane 8 versus lane 9*) and *D* and *E* (*lane 7 versus lane 4*)) and Fig. S13J (*lane 7 versus lane 8*)), and the combination of MA3* and ΔNCA clearly diminished ESCRT coimmunoprecipitation with Gag-GFP (Fig. 6 (*B* (*lane 9 versus lane 2*) and *D* and *E* (*lane 4 versus lane 2*)). For Bro1 binding, some additional reduction was visible, when p6 was deleted (Fig. S13J, *lane 8 versus lane 9*). Although we did not observe reduced Bro1 binding to Gag(V35E)-GFP, the Bro1 amount that coimmunoprecipitated with Gag(V35E/ΔNCA/Δp6)-GFP was reduced compared with Gag(ΔNCA/Δp6)-GFP (Fig. S13J, *lane 6 versus lane 3*). This finding indicates an effect of V35E when the Gag-Bro1 interaction is impaired by deleting NCA and p6.

Our results, with combined mutations in MA, CA, and p6, confirm the results of our binding studies with isolated Gag domains (Fig. 3). Our data suggest that Bro1 can bind to MA, NCA, and p6 and, furthermore, that Vps23 binds to MA and NCA.

### MA mutations that reduce Gag-ESCRT binding increase Gag-PM association and enhance Gag release from yeast

Our findings indicate that MA and NCA contribute to Gag-ESCRT binding. However, the MA3* and ΔNCA mutations caused contrary effects on Gag release. Yeast spheroplasts released a higher Gag(MA3*)-GFP amount compared with Gag-GFP and a lower Gag(ΔNCA)-GFP amount (Fig. 7*A*). ΔNCA produced a dominant negative effect over MA3* (Gag(MA3*/ΔΝCA)-GFP) (Fig. 7*A*, *S13I*). P6 deletion did not further reduce Gag(MA3*/ΔΝCA)-GFP release (Fig. S13I). To test whether the MA3* and ΔNCA effects may be caused by abnormal intracellular localization, assembly, or PM binding, we used fluorescence microscopy and prepared membrane sediments. Gag(MA3*)-GFP as well as Gag(ΔNCA)-GFP formed punctate structures at the PM similar to Gag-GFP, and we did not observe any intracellular aggregates (Fig. 7*C* and Figs. S14 and S15). The Gag(ΔNCA)-GFP amount that sedimented with membranes and the distribution pattern in the differential centrifugation were similar to Gag-GFP (Fig. 7 (*E* and *D*) and Fig. S13B). Thus, our experiments did not indicate impaired Gag(ΔNCA)-GFP assembly. A higher Gag(MA3*)-GFP amount compared with Gag-GFP distributed to the membrane containing 25,000 × *g* sediment (Fig. 7 (*D*, *E*, and *G*) and Fig. S13 (A–C)). This observation might explain increased Gag(MA3*)-GFP release. However, spheroplasts released a lower Gag(MA3*/ΔNCA)-GFP amount compared with Gag-GFP, even though a higher Gag(MA3*/ΔNCA)-GFP amount sedimented with membranes (Fig. 7 (*A* and *E*) and Fig. S13 (B and I)). Thus, stronger PM binding is not sufficient for increased Gag release when aa 126–277 are deleted.

To test whether increased Gag(MA3*)-GFP-PM binding, increased release, and reduced ESCRT binding are correlated, we examined membrane association and release of additional Gag-GFP versions with MA mutations in Leu-31, Val-35, and Trp-36. All mutants formed fluorescent punctate structures at the PM similar to Gag-GFP, and we did not observe any intracellular aggregates (Gag(MA3*)-GFP is shown as an example in Fig. S14). With the exception of Gag(V35E)-GFP, higher amounts of all mutants sedimented with membranes compared with Gag-GFP (Fig. 7*G* and Fig. S13 (A–C)). Moreover, unlike V35E, the tested mutations of Leu-31 and Trp-36 increased Gag-GFP release from spheroplasts (Fig. 7*F* and Fig. S13 (D–H)). In contrast to the other mutants, V35E did not reduce the Gag-ESCRT interaction in our coimmunoprecipitation experiments (see above). Thus, our results suggest that reduced Gag-ESCRT binding via MA, increased Gag-PM binding, and increased Gag release are correlated.

**Figure 8:**
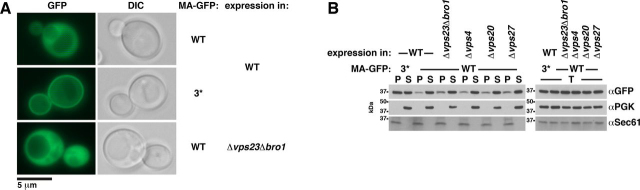
MA3* increases MA-GFP-PM binding, whereas ESCRT deletion does not.*A* and *B*, WT yeast cells or the indicated ESCRT mutants expressed MA-GFP or MA3*-GFP from a 2µ vector with induced MET3 promoter. *A*, MA-GFP binding to the PM was analyzed by fluorescence microscopy, showing PM rim staining for MA3*. *DIC*, differential interference contrast. *B*, membrane-containing 232,000 × *g* pellets (*P*) and cytosol-containing supernatants (*S*) derived from cell extracts (*T*) were analyzed by immunoblotting with anti-GFP antibodies, showing that increased MA3* amounts sediment compared with MA, whereas similar MA amounts sediment from WT and ESCRT mutant cell extracts. The pellet samples were 5× concentrated compared with the supernatant and extract samples. The cytosolic protein PGK and the integral ER membrane protein Sec61 served as references.

The inverse correlation between MA-PM binding and ESCRT interaction allows two interpretations: first, binding of ESCRT proteins or an ESCRT-recruiting factor to MA prevented MA-PM binding; or second, MA-PM binding replaced MA-bound ESCRT proteins or an ESCRT-recruiting factor. To test whether a potential direct ESCRT-MA binding may reduce Gag-PM interaction, we analyzed Gag-GFP– and MA-GFP–PM association in ESCRT knockout mutants. ESCRT deletion *(*Δ*bro1*, Δ*vps23*, Δ*vps23*Δ*bro1*, Δ*vps4*, Δ*vps27*) did not increase the Gag-GFP amount that sedimented with membranes (Fig. 2*E*). MA-GFP showed a cytosolic fluorescence, whereas we observed PM rim staining in addition to cytosolic fluorescence for MA3*-GFP (Fig. 8*A* and Figs. S16 and S17). This finding indicates that, similar to Gag(MA3*)-GFP, MA3*-GFP interacts stronger with the PM than the WT protein. When Δ*vps23*Δ*bro1* expressed MA-GFP, we observed only cytosolic staining (Fig. 8*A*). Consistent with these results, a higher MA3*-GFP proportion sedimented at high speed compared with MA-GFP, whereas the sedimented amounts of MA-GFP expressed in ESCRT deletion mutants (Δ*vps23*Δ*bro1*, Δ*vps4*, Δ*vps20*, Δ*vps27*) were similar to WT (Fig. 8*B*). Our results suggest that none of the corresponding ESCRT proteins prevented the MA-PM interaction. This state of affairs implies either that MA-PM binding replaced ESCRT proteins that were directly or indirectly bound to MA or that an ESCRT-recruiting factor, bound to MA, prevented MA-PM binding. Inasmuch as we proposed that ESCRTs may bind via their ubiquitin-binding domains (UBDs) directly to a hydrophobic patch in MA (see above), we cannot exclude the possibility that MA- and Gag-PM binding might be increased in a yeast strain with deletion of all ESCRT proteins containing UBDs.

### Increased Gag release caused by MA mutation is linked to ESCRT function

The increased Gag(MA3*)-GFP-PM binding and the reduced Gag(MA3*)-GFP-ESCRT interaction were both correlated with increased Gag(MA3*)-GFP release. Thus, the inverse correlation between MA-PM binding and MA-ESCRT interaction may be explained by two possible mechanisms. Possibly, the dissociation of an ESCRT-recruiting factor from MA enhances MA-PM interaction. Alternatively, Gag-PM binding conceivably replaces a direct or indirect MA-ESCRT interaction, which transiently blocked ESCRT function. We asked whether increased Gag(MA3*)-GFP release depends on ESCRT proteins or whether MA3* merely enhances the ESCRT-independent release by increasing the Gag-GFP amount at the PM. To test these possibilities, we then compared Gag-GFP and Gag(MA3*)-GFP release from a VPS4 deletion mutant, which completely blocks the ESCRT function (49, 50). Δ*vps4* abolished the increased release caused by MA3* (Fig. 9*A* and Fig. S18 (K and L)), with the exception of the first VLP harvest after 3 h of incubation, which, as we mentioned above, is partly ESCRT-independent. At this first time point recorded, the higher membrane-bound Gag(MA3*)-GFP amount may result in a stronger ESCRT-independent release. Release assays with knockout mutants of the early acting ESCRT factors Vps23 and Bro1, which can independently activate ESCRT-III (68), supported the finding that the MA3* release–increasing effect depends on ESCRT proteins. The Δ*vps23*Δ*bro1* mutant nearly abolished the MA3* release–increasing effect, whereas we observed increased Gag(MA3*)-GFP release from single knockouts (Fig. S18, A–J).

Gag(ΔNCA)-GFP release from yeast was reduced compared with Gag-GFP. Our binding studies suggested that yeast ESCRT proteins can bind to NCA. To test whether the Gag-GFP release reduction caused by ΔNCA was due to reduced ESCRT binding, we compared Gag-GFP and Gag(ΔNCA)-GFP release from Δ*vps4* spheroplasts (Fig. 9*B* and Fig. S18M). VPS4 deletion reduced Gag-GFP release stronger than ΔNCA. This finding may be explained by the fact that NCA does not carry the sole ESCRT-binding site in Gag. Inasmuch as ΔNCA reduced Gag-GFP release from Δ*vps4* spheroplasts even slightly further, this experiment does not allow the conclusion that the negative ΔNCA effect on Gag-GFP release was only caused by reduced ESCRT binding. ΔNCA may reduce VLP release by an additional or another mechanism.

### MA hydrophobic-patch mutation increases Gag release from HEK293 cells dependent on NCA and the ALIX-binding site in p6

Similar to yeast, MA3* increased Gag-GFP release from HEK293 cells (Fig. 9 (*D* and *E*), Fig. S18N, and Table 1). Gag(MA3*)-GFP release was strongly reduced by p6T*, which was similar to Gag-GFP. ΔNCA or p6A* did not impair Gag-GFP release (Fig. 9 (*D*, *F*, and *G*) and Fig. S18N). The impact of NCA and p6A on Gag release became evident in experiments involving Gag(MA3*)-GFP. A combination of ΔNCA and p6A* abrogated the increased Gag(MA3*)-GFP release (Fig. 9*D* (*lane 5 versus lanes 2* and *1*) and Fig. S18N). This result confirms our finding from yeast that the MA3* effect requires ESCRT proteins. Inasmuch as we observed binding of the yeast ESCRT proteins Bro1 and Vps23 to NCA in our coimmunoprecipitation experiments, it is tempting to speculate that ALIX may also interact with NCA, but evidence from binding studies is lacking. In contrast to human cells, we did not observe any p6 impact on Gag release from yeast, which is consistent with only weak Bro1 binding to p6 compared with the Gag N-terminal protein region and no visible Vps23 binding. Because ΔNCA reduces Gag-GFP release from yeast, which is in contrast its effect in HEK293 cells, the possibility remains that the ESCRT binding to NCA in yeast may take over the p6 function.

**Table 1:** Comparison of binding and Gag-GFP release assays with yeast and HEK293 cells

	Yeast		HEK293	
**Gag-GFP release:**				
MA3*	Increased		Increased	
Δp6	Efficient		Impaired	
p6T*	Efficient		Impaired	
p6A*	Efficient		Efficient (the combination of DNCA and p6A* abolished	
ΔNCA	Impaired		Efficient the MA3* release-increasing effect)	
	**Bro1**	**Vps23**	**ALIX**	**TSG101**
**Binding to:**				
GST-MA	Yes	Yes	Yes	Yes (stronger binding to GST-p6)
MA-GFP	Yes	Yes	Yes	
CA-GFP	Yes	Yes		
Gag(MA3*/ΔNCA)-GFP compared with Gag-GFP	Reduced	Reduced		
GST-p6	Yes	No	Yes	Yes
GagΔp6-GFP compared with Gag-GFP	Not reduced	Not reduced	Reduced	Reduced

We favor the following model to explain our results: ESCRT proteins can bind via MA, NCA, and p6 to Gag. Gag binding to the PM replaces an MA-ESCRT interaction and thereby activates ESCRT-dependent virion scission, which requires ESCRT binding to other sites in Gag.

Finally, the published ALIX interaction with NC (97, 104) was not considered in our experiments. A potential Bro1-NC interaction may require a lower salt concentration than that used in our binding studies.

## Discussion

HIV-1 budding through the PM depends on the viral protein Gag and the cellular ESCRT machinery. Two early acting ESCRT proteins, TSG101 and ALIX, bind to the Gag C-terminal p6 peptide (23, 24, 25, 26, 83, 84). The TSG101-binding site is important for virion release. The role of the ALIX-binding site is enigmatic because its mutation has only mild effects. Steps following early factor recruitment are poorly understood. For proper viral particle composition, Gag assembly and ESCRT action must be coordinated. Since ESCRT discovery, yeast has been a powerful model to study ESCRT function. The yeast ESCRT machinery consists of single-protein versions that can be analyzed with viable knockouts. Thus, a yeast model could help to elucidate the viral budding mechanisms, without interfering effects of antiviral factors present in human cells. Previous work reported HIV-1 Gag-VLP release by yeast spheroplasts (86), but Gag release by the published protocol was ESCRT-independent (87). We developed a different protocol and showed by using ESCRT knockout mutants that yeast ESCRTs determine Gag release. Moreover, binding assays indicated that Vps23 and Bro1, the yeast TSG101 and ALIX homologs, were in physical contact with Gag. We followed Gag release over 7 h after spheroplast preparation. Norgan *et al*. (87) described ESCRT-independent Gag release after incubating spheroplasts for 2 h. The short incubation time might explain why the authors did not observe ESCRT-dependent release. In our assay, Gag release at the first harvest after 3 h was partly ESCRT-independent. Over the following time period, Gag release increased and was strongly ESCRT-dependent. Gag released during the first incubation period might be mainly derived from VLPs that had preassembled beneath the intact cell wall. Particle formation delay caused by an ESCRT-independent mechanism might be not apparent in this case, as these particles could not be released until cell wall removal. Sakuragi *et al*. (86) showed a decreasing Gag release over a longer incubation time, which is in contrast to the increasing release that we observed. Thus, additional differences in the protocol may be important to enable ESCRT-driven Gag release.

We used our yeast system to characterize the interaction of Gag mutants with genomically epitope-tagged ESCRT proteins and to analyze Gag release and PM binding with ESCRT knockout strains. We identified a previously unknown ESCRT interaction with the Gag N-terminal protein region. Binding assays indicated Bro1 and Vps23 interacting with MA and NCA. Moreover, we confirmed the MA interaction for the human homologs, ALIX and TSG101. Functional data suggest that ALIX may also interact with NCA (see below). In contrast to HIV-1, several other retroviral Gag proteins carry ESCRT-binding motifs not at the C-terminal end, but in their N-terminal half (reviewed in Ref. 105). Together with our findings, this fact may indicate that ESCRT binding to the Gag N-terminal protein region is generally important for the release mechanism. The shorter distance to the PM compared with the described C-terminal ESCRT-binding sites allows a new view on the mechanism that ESCRTs may use to assist viral particle formation.

We identified a hydrophobic patch consisting of Leu-31, Val-35, and Trp-36 on the MA globular head surface, the mutation of which reduced the Bro1 and Vps23 amount that coimmunoprecipitated with MA. As we performed our binding assays with cell extracts, other cellular factors may be involved in the interaction. We excluded the possibility that Bro1 or Vps23 was recruited via the other protein to MA by using knockout mutants. The observation that the identified MA hydrophobic patch is similar to the ubiquitin hydrophobic patch, which mediates the interaction with UBDs of early acting ESCRT proteins, including Bro1, Vps23, and their human homologs (37, 55, 56, 57, 58, 63, 67), could explain the direct binding of both proteins. Our results further indicated that the MA-ESCRT interaction is transient and is replaced when Gag binds to the PM. MA hydrophobic patch mutations that reduced Gag-ESCRT binding increased Gag-PM binding. This finding is consistent with the observation that the hydrophobic patch is located on the MA site that faces the PM. We observed increased Gag-PM binding for Leu-31 and Trp-36 mutations. Based on X-ray data (11), the hydrophobic patch is not part of the trimerization interface and probably the mutations do not enhance myristoyl chain exposure by increasing MA trimer formation. Moreover, two NMR studies, characterizing myristate exposure, do not describe Leu-31 or Trp-36 contact with the sequestered myristoyl chain (18, 106). Inasmuch as both L31R and L31E increased Gag-PM binding, increased affinity to negatively charged phospholipids is unlikely to be the reason for enhanced PM binding by Leu-31 mutation. Increased release of HIV-1 carrying Gag(W36A) has been described (107). Whether Trp-36 interacts with an extended lipid acyl chain as proposed in an NMR study is controversial (19, 99), and whether the Trp-36 mutations used in our study could increase membrane binding by affecting the proposed interaction is unclear. Additional techniques are necessary to clarify the question of whether the Gag mutants used in our study directly increase PM affinity or whether the decreased affinity to an MA binding factor enables increased PM binding. Because Gag-PM and MA-PM binding in several ESCRT knockout mutants were not increased compared with WT, we assumed that a potential direct MA binding of the corresponding ESCRT proteins did not prevent the Gag-PM interaction.

The fact that p6 can recruit human ESCRT proteins raises the question of why a transient ESCRT interaction with MA is useful for virus release. MA mutations that reduced Gag-ESCRT binding increased Gag release, which is in contrast to p6 deletion in human cells. Two experiments indicated that the release-increasing effect was ESCRT-coupled: VPS4 deletion abrogated increased Gag(MA3*) release from yeast and a combination of ΔNCA and a mutated ALIX-binding site in p6 (p6A*) from HEK293 cells. The latter finding was especially remarkable because Gag(p6A*) and Gag(ΔNCA) were efficiently released. Our findings from yeast and human cells suggest that the ESCRT-MA interaction blocks ESCRT function until the interaction is replaced by Gag-PM binding. Our results also help to explain the enigmatic role of the ALIX-binding site in p6. Our GST-pulldown experiments showed that the relative affinity for MA compared with p6 is higher for ALIX than for TSG101. Assuming that ESCRT proteins require binding to p6 while promoting virus release, the reduced interaction with MA3* would especially affect the ALIX function. As the combination of ΔNCA and p6A* abolished the MA3* effect, it is tempting to speculate that ALIX also acts via binding to NCA, although evidence for CA interaction with human ESCRT proteins from binding assays is lacking. Consistent with this model, p6A mutations strongly impaired release of HIV-1 carrying a minimal Gag construct, Gag(Δ8-87/Δ126-277), lacking the MA globular head and NCA (26). Our assumptions suggest that ALIX interacts with several sites in Gag and that its activity may be regulated by Gag assembly. An additional RNA- or membrane-dependent binding to the NC domain has been described (108). ALIX function can be regulated by switching between an open and a closed conformation and by dimerization, and moreover, a regulatory function for Bro1 and its binding partner Doa4 has been proposed (109, 110, 111, 112, 113).

In contrast to Gag-GFP release from HEK293 cells, Gag-GFP release from yeast did not require p6. Consistent with these results, we observed only weak Bro1-p6 binding and no Vps23-p6 interaction. In contrast to HEK293 cells, ΔNCA reduced WT Gag release from yeast. Thus, it is conceivable that in yeast, the NCA-ESCRT binding may take over the p6 function, although we cannot exclude other potential ΔNCA effects. According to a computational simulation, NCA contributes to particle curvature and certain NCA point mutations block proper membrane bending and virus release (114, 115, 116). However, Gag assembly may be sensitive to NCA composition when NCA is present. *In vitro* CCA can assemble an immature-like lattice independent of NCA (21), and we did not find any hint of impaired Gag(ΔNCA) assembly in yeast. The combination of our results suggests that NCA may be involved in functional Gag-ESCRT interaction. Inasmuch as yeast ESCRT proteins bound to CA in coimmunoprecipitation experiments but not to GST-CA expressed in *E.coli*, an NCA posttranslational modification, namely ubiquitination, may be required for the interaction. Gag ubiquitination at multiple sites distributed over the whole molecule involved in HIV-1 release has been described (117, 118, 119).

Gag conformational changes during assembly from a compact folding to an extended structure (4, 5) may contribute to dynamic changes of the Gag-ESCRT interplay. The transient MA-ESCRT binding may help to block ESCRT-III polymerization until Gag is sufficiently assembled for virion abscission. This model is consistent with microscopic studies for HIV-1 and EIAV (equine immunodeficiency virus) Gag-VLP release, which indicate that the late acting ESCRT-III proteins and VPS4 are recruited when Gag assembly is completed, whereas the early acting ESCRT factors ALIX and TSG101 are recruited along with Gag (90, 120). Evidence that ESCRT-mediated scission controls virion composition indicates that coordination of ESCRT activation with Gag assembly has an implication for infectious-virion generation. Gag overpolymerizes in budding-arrested virions, suggesting that ESCRT-mediated release occurs in kinetic competition with Gag polymerization (73). Delayed particle neck scission by disrupted ESCRT-p6 interaction leads to enzyme leakage through the open particle neck (85). Premature scission could also generate incomplete particles (*e.g.* a certain Gag molecule number is required for capsid formation, MA incorporates viral envelope proteins, and cellular membrane proteins protecting the virus from the innate immune response are sorted into the viral envelope) (121, 122, 123).

The finding that ESCRT-0 subunit VPS27 deletion reduced Gag release was unexpected, as Vps27 binds to the endosomal membrane via a FYVE domain (124). Thus, it remains to be clarified whether Gag can recruit ESCRT-0 to the PM. ESCRT-0 contains several UBDs (37, 69, 125) that might interact with potential Gag ubiquitin modifications or the MA hydrophobic patch. Data from human cells can be interpreted to indicate that, besides being required to suppress the antiviral activity of tetherin, the Vps27 homolog HRS may have an additional function for HIV-1 release (126).

Our yeast system revealed a previously unknown Gag-ESCRT interaction with a conceivable impact for the budding mechanism. This presumably transient interaction became apparent in the yeast system because the ESCRT-p6 interaction was less prominent than in human cells. Thus, although, or even because not matching completely, yeast offers a complementing tool to study the basic Gag budding mechanism. Comparing differences and similarities may further contribute to better understanding the precise role of the individual interactions and steps during the budding process. Moreover, yeast genetics allows a kind of functional analysis that is not easily available with other systems. Comparing yeast and human ESCRT proteins interacting with the same budding substrate, Gag, may additionally help us learn more about the ESCRT mechanism in general.

## Experimental procedures

### Yeast strains

Yeast strains used in this study are listed in Table S1. They were derived from YWO1, a haploid DF5 descendent. If not indicated differently in the figure legends, YWO1 served as WT. For gene deletion and chromosomal epitope tagging, we followed methods described previously (127, 128). To test whether epitope-tagged ESCRT proteins were functional, we analyzed the transport and processing of the MVB substrate GFP-CPS by immunoblotting (31, 129) (Fig. S19). The vector GFP-CPS 416 was a gift of Hugh Pelham.

### Plasmid construction

The templates for Gag-GFP and Gag(p6T*)-GFP coding sequence amplification (pGag-EGFP, pGagLTAL-EGFP) were gifts of Marylin Resh (130). We amplified the coding sequences by PCR with primers containing appropriate restriction sites and constructed the mutant Gag-GFP versions by PCR mutagenesis. For expression in yeast, we subcloned the coding sequences into pYPGE2 (PGK promoter, CYC1 termination signal) or pRS425 and pRS415 with MET3 promoter and CYC1 termination signal. The MET3 promoter and CYC1 termination signal sequences were as described (131). To gain sufficient Gag-GFP expression from pEGFP-N1 (Clontech) in HEK293 cells, we subcloned 38 bp upstream of the ATG in pGag-EGFP in front of the ATG in pEGFP-N1. To express GST-tagged Gag domains in *E. coli*, we used pGEX-6-P1. All constructs were verified by sequencing. *E. coli* strains XL1-blue and DH5 α were used for cloning. We purchased plasmids for the expression of epitope-tagged ALIX and TSG101 from Gene Copoeia (pReceiver vector).

### Antibodies

Antibodies against Sec61 were as described (132). H. D. Schmitt provided anti-Emp47 antibodies. We derived monoclonal anti-HA antibodies from hybridoma cells (12CH5). We purchased anti-PGK (A6457, Molecular Probes), anti-GST (600-101-200, Rockland), polyclonal anti-GFP for immunoprecipitation (A6455, Molecular Probes), monoclonal anti-GFP for immunodetection (JL-8, Clontech), anti-Myc (M5546, Sigma–Aldrich), anti-FLAG (M2, F3165, Sigma–Aldrich), and anti-GAPDH (MAB374, Sigma–Aldrich). Horseradish peroxidase–coupled secondary antibodies (Sigma–Aldrich) were used to visualize immunoblots by Western Lightning Plus ECL (PerkinElmer Life Sciences) and autoradiography films (X-Omat, Kodak). For immunogold labelings, a rabbit anti-GFP antibody (Abcam, ab6556; diluted 1:100) and a 12-nm colloidal gold goat anti-rabbit secondary antibody (Dianova, 111-205-144; diluted 1:30) were used.

### Yeast experiments

In all experiments, yeast cells were grown at 30 °C and harvested in their exponential growth phase. The OD_600_ was measured with an Ultrospec 3000 photometer (Pharmacia Biotech). We transformed yeast with expression vectors using the standard lithium acetate method. Afterward, cells were grown in standard synthetic dextrose (SD) minimal medium supplemented with essential amino acids. For yeast extract preparation in GST-pulldown experiments, yeast cells were grown in standard yeast extract peptone dextrose (YPD) complete medium.

Before protein extraction, we washed yeast cells with ice-cold 10 mm sodium azide and performed the following steps on ice if not indicated differentially. We centrifuged samples at 4 °C. For centrifugations at 232,000 × *g*, the TLA100.3 rotor was used. Prior to SDS-PAGE, proteins were dissolved in sample buffer by heating at 90 °C.

### Whole-cell extract preparation

Yeast cells were disrupted with glass beads in 50 mm Tris (pH 7.5) and 1% SDS containing protease inhibitors. Lysates were adjusted to 50 mm Tris (pH 7.5), 0.1% SDS, 1% Triton X-100, 150 mm NaCl, and 5 mm EDTA, removed from glass beads, and cleared by centrifugation (10 min, 16,000 × *g*). The supernatant was subjected to TCA precipitation.

### Membrane and cytosol preparation

Yeast cells were resuspended in 50 mm Tris (pH 7.5) and 10 mm EDTA containing protease inhibitors and lysed with glass beads. Cell lysates were removed from glass beads with buffer and cleared by low-speed centrifugation (400 × *g*, 10 min). An aliquot was diluted with the same volume of 2× sample buffer (total). To prepare membranes, the extract was centrifuged (25,000 × *g*, 30 min for Gag-containing pellets or 232,000 × *g*, 30 min for MA-containing pellets). The cytosol-containing supernatant was diluted with the same volume of 2× sample buffer. The pellet was resuspended in the 2-fold volume of sample buffer, if not indicated differentially.

### Differential centrifugation

Yeast cells were resuspended in 50 mm Tris (pH 7.5) and 10 mm EDTA containing protease inhibitors and lysed with glass beads. Cell lysates were removed from glass beads with buffer and cleared by low-speed centrifugation (400 × *g*, 10 min). An aliquot was diluted with the same volume of 2× sample buffer (total). The supernatant was subjected to differential centrifugation, first 25,000 × *g* for 30 min and the resulting supernatant at 232,000 × *g* for 30 min. The pellets of both centrifugation steps were resuspended in 2× sample buffer, and the supernatant of the second centrifugation was diluted with the same volume of 2× sample buffer.

### Coimmunoprecipitation (yeast)

For each immunoprecipitation, 15 OD_600_ yeast cells were harvested. Cells were resuspended in 50 mm Tris (pH 7.5), 150 or 400 mm NaCl, and 10% glycerol containing protease inhibitors and lysed with glass beads. Proteins were extracted with 50 mm Tris (pH 7.5), 150 or 400 mm NaCl, 1% Triton X-100, and 10% glycerol containing protease inhibitors by rotating for 30 min at 4 °C. Debris and insoluble material were removed by centrifugation steps (400 × *g* for 10 min and 2× 13,000 × *g* for 10 min). Aliquots were taken and diluted with 2× sample buffer (input). GFP-tagged proteins were immunoprecipitated from the extract with polyclonal anti-GFP antibodies and Protein A–Sepharose (GE Healthcare) by rotating at 4 °C overnight. The beads were washed three times in extraction buffer and afterward eluted in sample buffer.

### Gag release assay (yeast)

After harvesting, yeast cells were washed in water. Spheroplasts were prepared from 5 OD_600_ cells by incubating the cells with 10 mm DTT and 50 mm Hepes (pH 7.5) for 10 min at 30 °C, removing DTT by washing the cells two times with 1 m sorbitol, and incubating the cells with Zymolyase 20T (0.3 mg/ml, MP Biomedicals) in SD medium buffered with 50 mm Hepes (pH 7.5) containing 1 m sorbitol for 30 min. To follow the progress of cell wall disruption, aliquots were resuspended in water, and burst of spheroplasts was detected by measuring the OD_600_. Zymolyase was afterward removed by washing the spheroplasts two times in SD medium containing 1 m sorbitol. Spheroplasts were incubated in SD medium containing 1 m sorbitol for the indicated times, and VLPs were harvested from the medium. Medium was taken after careful spheroplast sedimentation by using the short spin button of the table centrifuge and filtrated (0.45-μm pore size, cellulose acetate, Whatman/GE Healthcare) to remove debris. VLPs were collected by high-speed centrifugation (232,000 × *g*, 30 min), and the pellet was resuspended in sample buffer. At the last time point, the spheroplasts were lysed in sample buffer.

### Gag-membrane binding after Gag expression induction

Yeast cells were grown in SD medium containing 20 mg/liter methionine to repress Gag expression from the MET3 promoter. After harvesting, cells were washed in water and afterward concentrated to 4 OD_600_/ml SD medium lacking methionine. Aliquots were taken at the indicated times, and membranes, cytosol, and a total were prepared.

### HEK293 cell experiments

HEK293 Tet-On cells (Clontech) were cultivated in Dulbecco's modified Eagle's medium with 4.5 g/liter glucose (E15-810 PAA) and supplemented with 10% fetal calf serum (PAA). For transfection, cells were seeded into 6-well plates and transfected with the indicated expression vectors at the following day using Lipofectamine LTX (Invitrogen) or Lipofectamine 2000 (Invitrogen) according to the manufacturer's instructions. We analyzed the cells 2 days after transfection. Cells were harvested by scraping in PBS containing protease inhibitors and centrifugation (700 × *g*, 5 min). If not otherwise indicated, samples were handled on ice, and centrifugations were performed at 4 °C. For centrifugations at 232,000 × *g*, the TLA100.3 rotor was used. Prior to SDS-PAGE, proteins were dissolved in sample buffer by heating at 90 °C.

### Coimmunoprecipitation (HEK293)

For each immunoprecipitation, the cell lysate of one well of a 6-well plate was used. Cells were lysed by rotating at 4 °C for 30 min in 50 mm Tris (pH 7.5), 150 or 400 mm NaCl, 1% Triton, 10% glycerol, and protease inhibitors. Debris and insoluble material were removed by centrifugation (1,000 × *g* for 10 min and two times at 13,000 × *g* for 10 min). An aliquot was taken (input), and GFP-tagged proteins were immunoprecipitated from the extract with polyclonal anti-GFP antibodies and Protein A–Sepharose by rotating at 4 °C overnight. The beads were washed three times in extraction buffer and afterward resuspended in sample buffer.

### Gag release assay (HEK293)

2 days after transfection, VLPs were harvested from the medium of one well of a 6-well plate. The medium was filtrated (0.45-μm pore size, cellulose acetate, Whatman/GE Healthcare) to remove debris. VLPs were collected by high-speed centrifugation (232,000 × *g*, 30 min), and the pellet was resuspended in sample buffer. For cell lysate preparation, cells were disrupted by incubation with 50 mm Tris (pH 7.5), 1% Triton, 0.1% SDS, 5 mm EDTA, and protease inhibitors for 15 min on ice. Debris was removed by centrifugation at 16,000 × *g* for 10 min. The supernatant was subjected to TCA precipitation.

### GST-pulldown assay

To express GST-fused proteins or peptides, BL21 *E. coli* cells transformed with appropriate plasmids were grown at 37 °C in Luria–Bertani medium with the addition of antibioticum for plasmid selection to 0.6 OD_600_. Afterward, cells were incubated overnight at 16 °C in the presence of 1 mm isopropyl 1-thio-β-d-galactopyranoside and 2% ethanol. After harvesting, cells were resuspended in PBS containing 5 mm EDTA and protease inhibitors and disrupted by sonication. Debris was removed by centrifugation (two times at 16,000 × *g* for 13 min). 1 mg/ml BSA and GSH-Sepharose (GE Healthcare) were added, and the samples were rotated for 1 h at 4 °C. 20 μl of GSH-Sepharose were loaded with the extract of 120 OD_600_ *E. coli* cells. Afterward, the beads were washed three times in PBS with the addition of 1% Triton X-100, 200 mm NaCl, 1 mg/ml BSA, and protease inhibitors. After washing, the beads were rotated with either yeast or HEK293 cell extract overnight. The beads were washed three times in incubation buffer and eluted with sample buffer. Yeast and HEK293 cell extracts were prepared as for coimmunoprecipitation experiments, and 1 mg/ml BSA was added before incubation with Sepharose. Extract of 3.4 OD_600_ yeast cells or extract of HEK293 cells derived from one well of a 6-well plate was loaded onto the beads.

### Fluorescence microscopy

GFP-tagged proteins were expressed as described in the figure legends. Yeast cells were concentrated by centrifugation and analyzed *in vivo* using an Axioplan II Zeiss microscope with a ×63 oil immersion objective, equipped with an Axiocam digital camera and Axiovison 4 software, using appropriate filter setups. Image processing was done using Adobe Photoshop. Images of representative cells are presented in the main paper, as demonstrated by figures in the supporting information showing a larger number of cells.

### EM

Fixation and immunolabeling were performed according to Ref. 133. Briefly, yeast cells were fixed with 4% freshly prepared formaldehyde, 0.5% glutaraldehyde (EM-grade) in 0.1 m sodium citrate buffer (pH 5.0) for 1 h at room temperature. After washing, samples were treated with 1% sodium *meta*-periodate for 1 h, followed by infiltration with 1.6 m sucrose, 25% polyvinylpyrrolidone K15 and frozen. Ultrathin cryosections according to Ref. 134 were labeled with anti-GFP antibody and 12-nm colloidal gold secondary antibody. Sections were contrasted and stabilized with a mixture of 3% tungstosilicic acid hydrate and 2.5% polyvinyl alcohol.

For plastic embedding, yeast cells were fixed with 1% glutaraldehyde in 0.1 m sodium citrate buffer for 1 h at 30 °C, followed by 4 °C overnight. After washing, samples were treated with 1% sodium *meta*-periodate for 1 h, followed by embedding the pellets in 10% gelatin in 0.1 m sodium cacodylate buffer. Samples were postfixed with 1% OsO_4_ for 2 h, dehydrated in a graded ethanol series and propylene oxide, and embedded in Poly/Bed® 812 (Polysciences, Inc., Eppelheim, Germany). Ultrathin sections were contrasted with uranyl acetate and lead citrate.

## Data availability

All data are included in the article and in the supporting information.
