# Supplementary material for: HIV-1 Gag release from yeast reveals ESCRT interaction with the Gag N-terminal protein region
Source: J Biol Chem. 2021 Jan 13;295(52):17950–72. doi: 10.1074/jbc.RA120.014710 (PMC7939435; doi:10.1074/jbc.RA120.014710)
Supplement: Supplementary file 1 [file mmc1.pdf]

JBC 2020-014710 revised

HIV-1 Gag release from yeast reveals ESCRT interaction with the Gag N-terminal protein region

Birgit Meusser, Bettina Purfuerst, Friedrich C. Luft

Supporting information

Fig. S1 shows that Gag-GFP forms punctate structures at the plasma membrane, visible by fluorescence microscopy.
Fig. S2 shows that Gag-GFP forms punctate structures at the plasma membrane, visible by fluorescence microscopy.
Fig. S3 shows that Gag(G2A)-GFP does not form punctate structures at the plasma membrane, visible by fluorescence microscopy.
Fig. S4 shows that Gag-GFP expression induces buds at the PM, visible by EM of Epon embedded yeast.
Fig. S5 shows that Gag-GFP expression induces buds at the PM, visible by EM of Epon embedded yeast.
Fig. S6 shows that Gag-GFP accumulates in PM buds of the WT YWO1 and SUB62, visible by EM of cryosections labeled with immunogold.
Fig. S7 demonstrates that ESCRT deletion does not impair Gag-GFP-membrane binding after MET3 promoter induction, that Gag-GFP expressed from a 2μ vector with MET3 promoter is released from yeast spheroplasts, that release depends on Gag myristoylation, is reduced by ESCRT deletion, that we did not detect release when Gag-GFP was expressed from the ARS/CEN vector, and demonstrates with additional experiments that release of Gag-GFP expressed from the 2μ vector with PGK promoter is reduced by ESCRT deletion.
Fig. S8 coimmunoprecipitation experiments, showing that Bro1 binds to CA, that Vps23 binds to NC-containing Gag fragments in the presence of 150 mM NaCl, that the presence of 400 mM NaCl does not alter the p6-dependent Gag binding to ALIX and TSG101 compared to 150 mM NaCl.
Fig. S9 binding assays comparing different epitope tags.
Fig. S10 shows that Gag(Δ8-87)-GFP accumulates at the PM in structures with larger diameters than Gag-GFP, visible by fluorescence microscopy.
Fig. S11 shows examples of coimmunoprecipitation experiments done to characterize the MA-Bro1 interaction.
Fig. S12 shows that Vps23 binding to MA is independent of Bro1 and vice versa.
Fig. S13 shows that L31 or W36 mutation increases Gag-GFP-membrane binding and Gag-GFP release from yeast spheroplasts, that the ΔNCA mutation does not reduce Gag-GFP-membrane association and reduces Gag-GFP release, is dominant over MA3*, that p6 deletion does not further reduce Gag(MA3*/ΔNCA)-GFP release, and that V35E reduces Bro1 binding to Gag-GFP in presence of ΔNCA and p6.
Fig. S14 shows that Gag(MA3*)-GFP forms punctate structures at the PM similar to Gag-GFP and does not form aggregates, visible by fluorescence microscopy.
Fig. S15 shows that Gag(ΔNCA)-GFP forms punctate structures at the PM similar to Gag-GFP and does not form aggregates, visible by fluorescence microscopy.
Fig. S16 shows PM rim staining of MA3*-GFP, visible by fluorescence microscopy.
Fig. S17 shows PM rim staining of MA3*-GFP, visible by fluorescence microscopy.
Fig. S18 shows that the MA3* release increasing effect depends on yeast ESCRT proteins, that Gag(ΔNCA)-GFP release from *Δvps4* spheroplasts is slightly decreased compared to Gag-GFP, that the MA3* mutation increases Gag-GFP release from HEK293 cells and that a combination of ΔNCA and mutation of the ALIX binding site in p6 (p6A*) abrogates this increase.
Fig. S19 shows that the genomically epitope-tagged ESCRT proteins used in this study are functional.
Table S1 lists yeast strains used in this study.

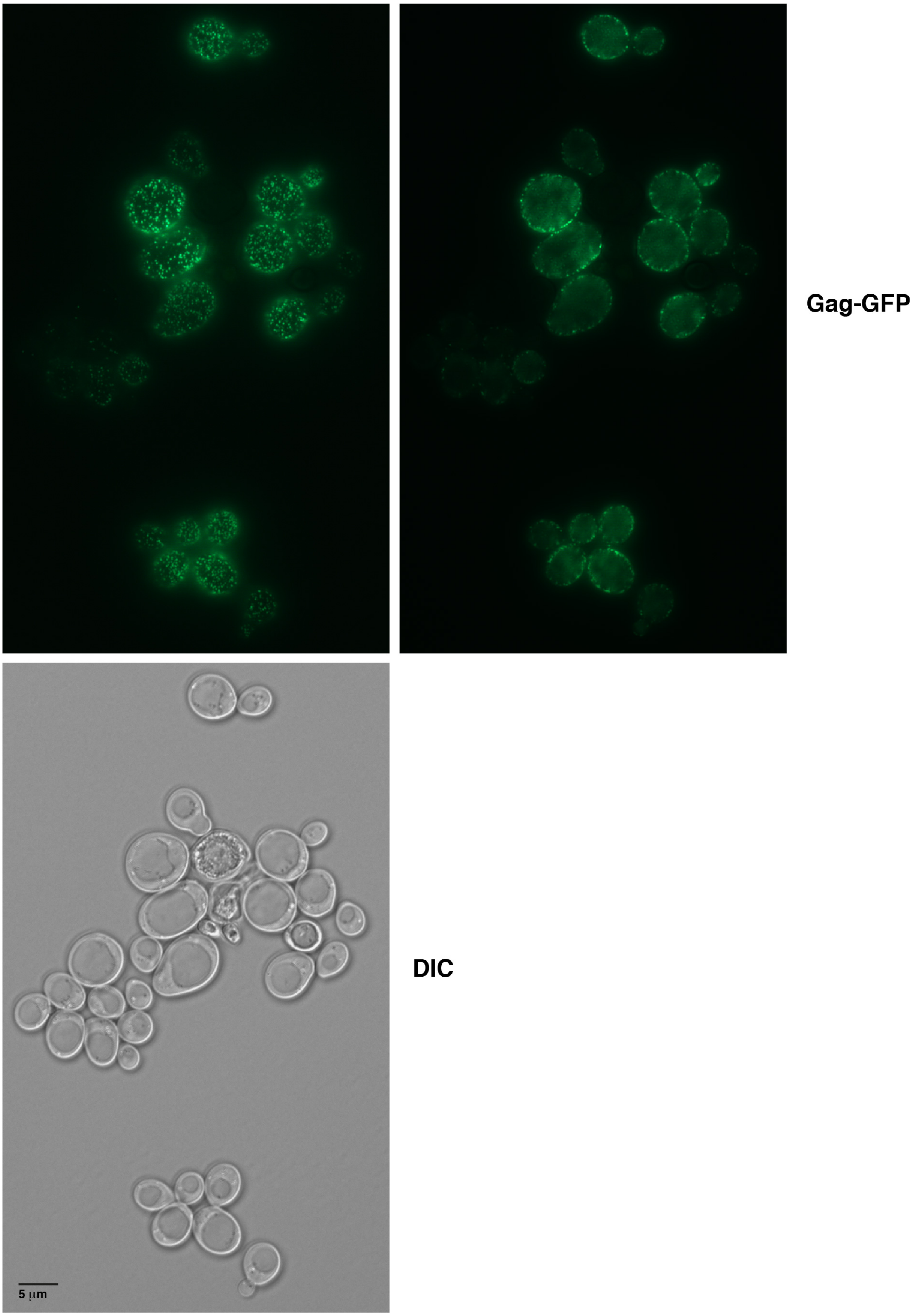

Fig. S1: The intracellular localization of Gag-GFP expressed from a 2µ vector with PGK promoter in WT yeast cells was analyzed by fluorescence microscopy, showing that Gag-GFP accumulates in punctate structures at the PM. Two layers of yeast cells are shown. DIC: differential interference contrast.

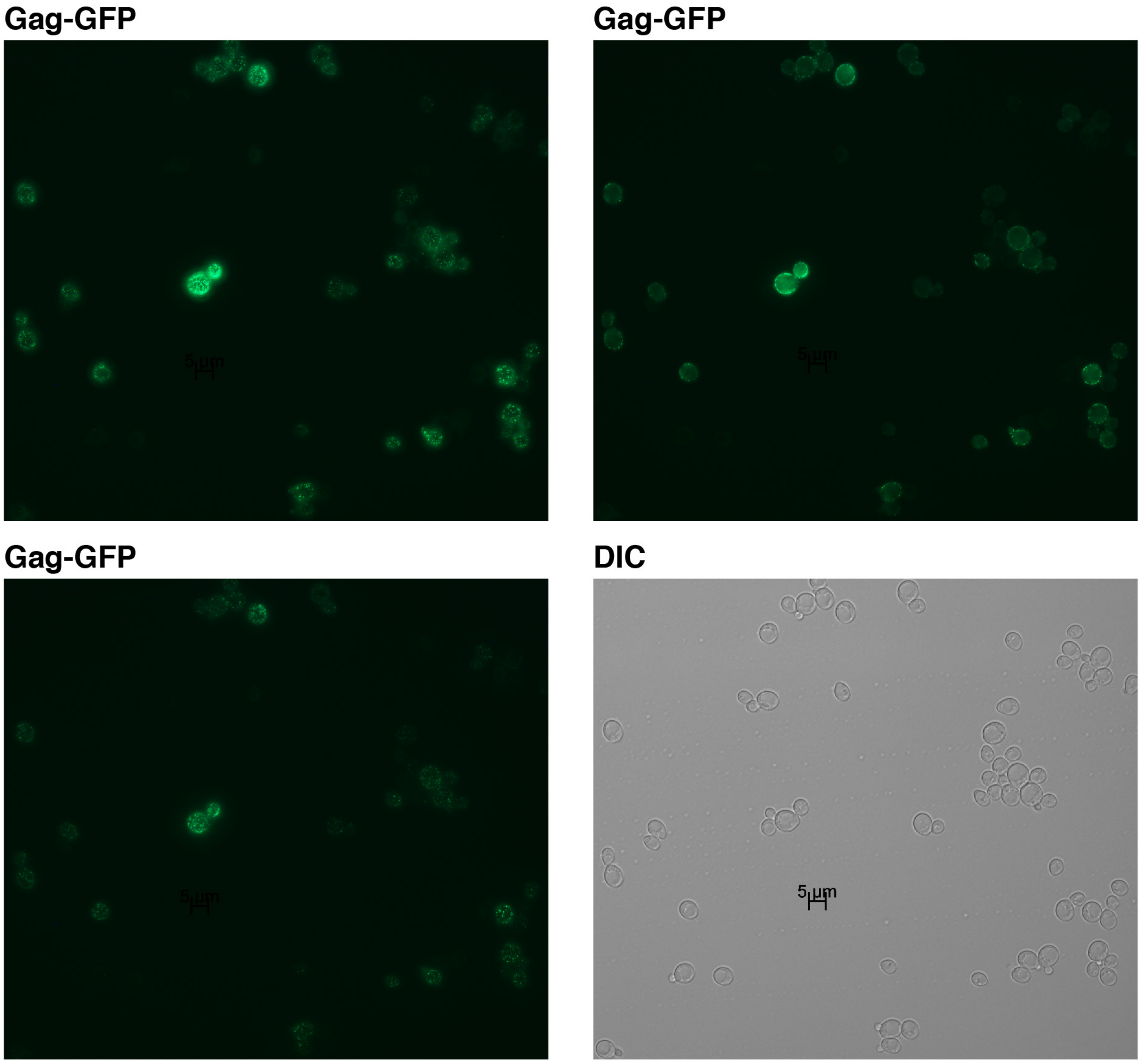

Fig. S2: The intracellular localization of Gag-GFP expressed from a 2μ vector with PGK promoter in WT yeast cells was analyzed by fluorescence microscopy, showing that Gag-GFP accumulates in punctate structures at the PM. Three layers of yeast cells are shown. DIC: differential interference contrast.

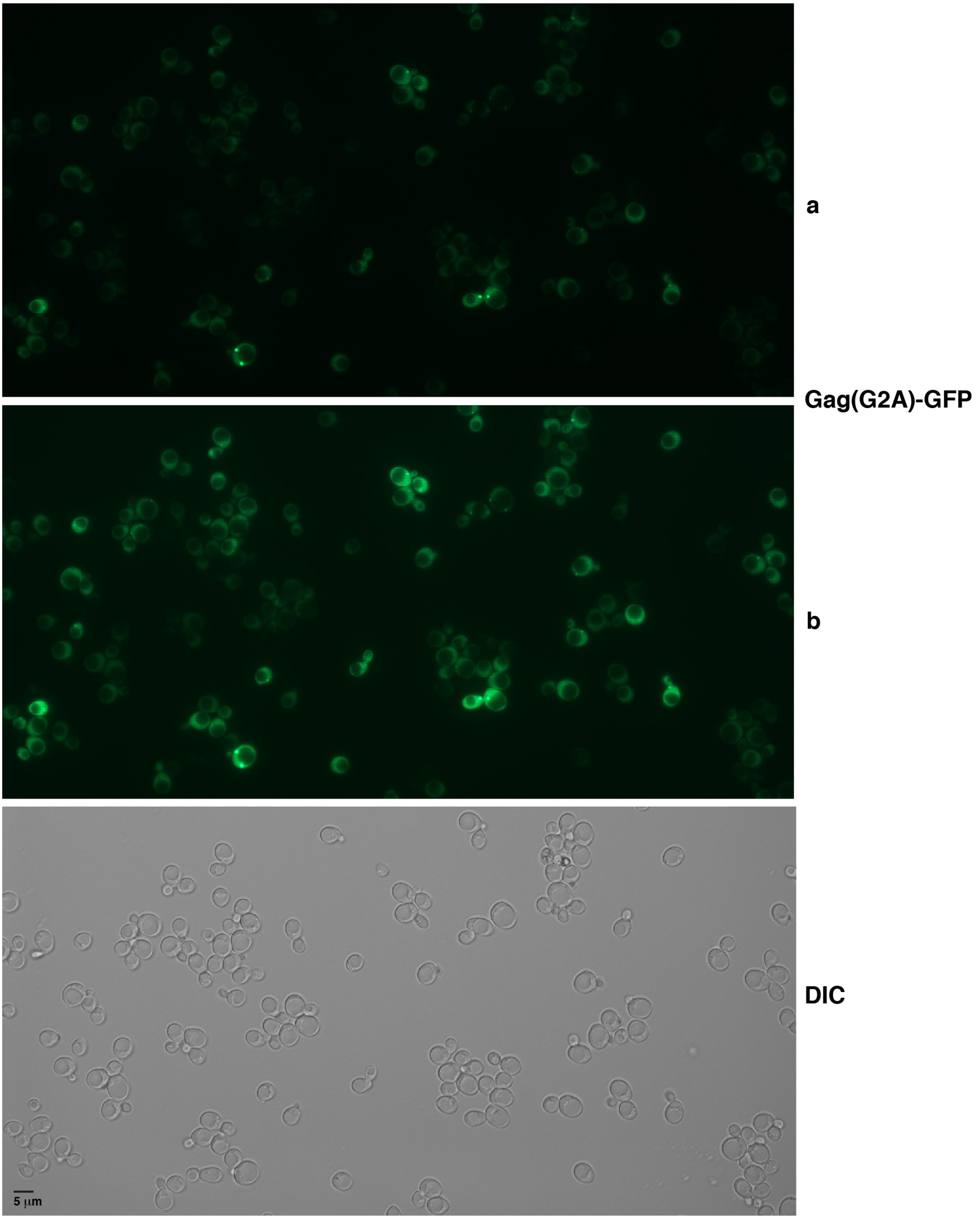

Fig. S3: Gag(G2A)-GFP expressed from a 2µ vector with PGK promoter in WT yeast cells was analyzed by fluorescence microscopy, showing a cytosolic localization. Gag(G2A)-GFP accumulations in undefined structures were visible in approximately one third of the cells. Enhanced brightness (b). DIC: differential interference contrast.

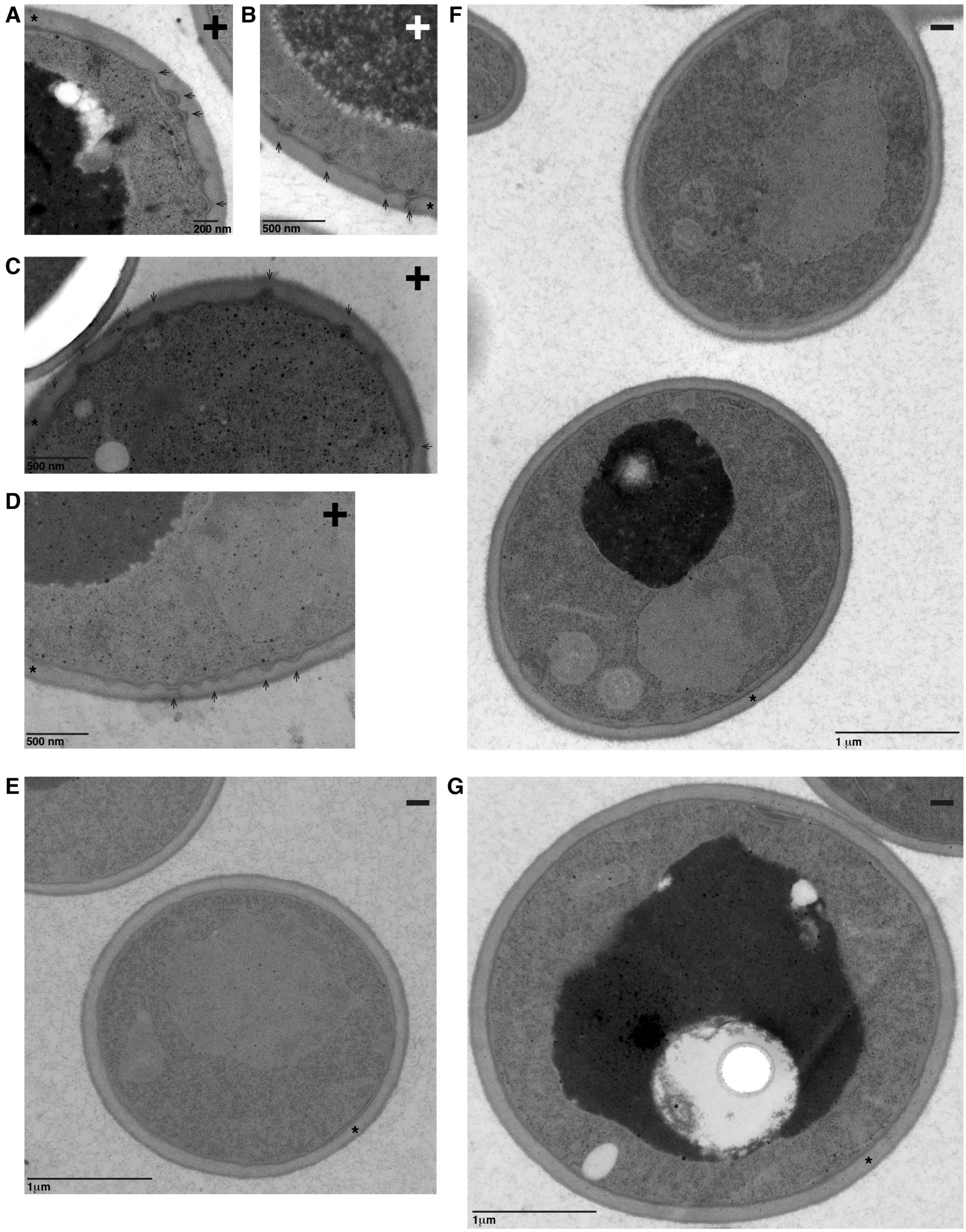

Fig. S4: WT yeast cells expressing Gag-GFP from a 2μ vector with MET3 promoter (+, **A.-D.**) or carrying the empty vector (-, **E.-G.**) were embedded in Epon. Sections were analyzed by EM, exhibiting buds (arrows) at the PM in presence of Gag-GFP. Cells were grown in medium containing 20 mg/l methionine to repress the promoter. To induce the promoter, cells were shifted to medium lacking methionine for 6 h. Sections shown in A, B, C, F, G are derived

from the same experiment. A. is also shown in Fig. 1F. Sections shown in D and E are derived from a second independent experiment. Asterisks indicate the cell wall.

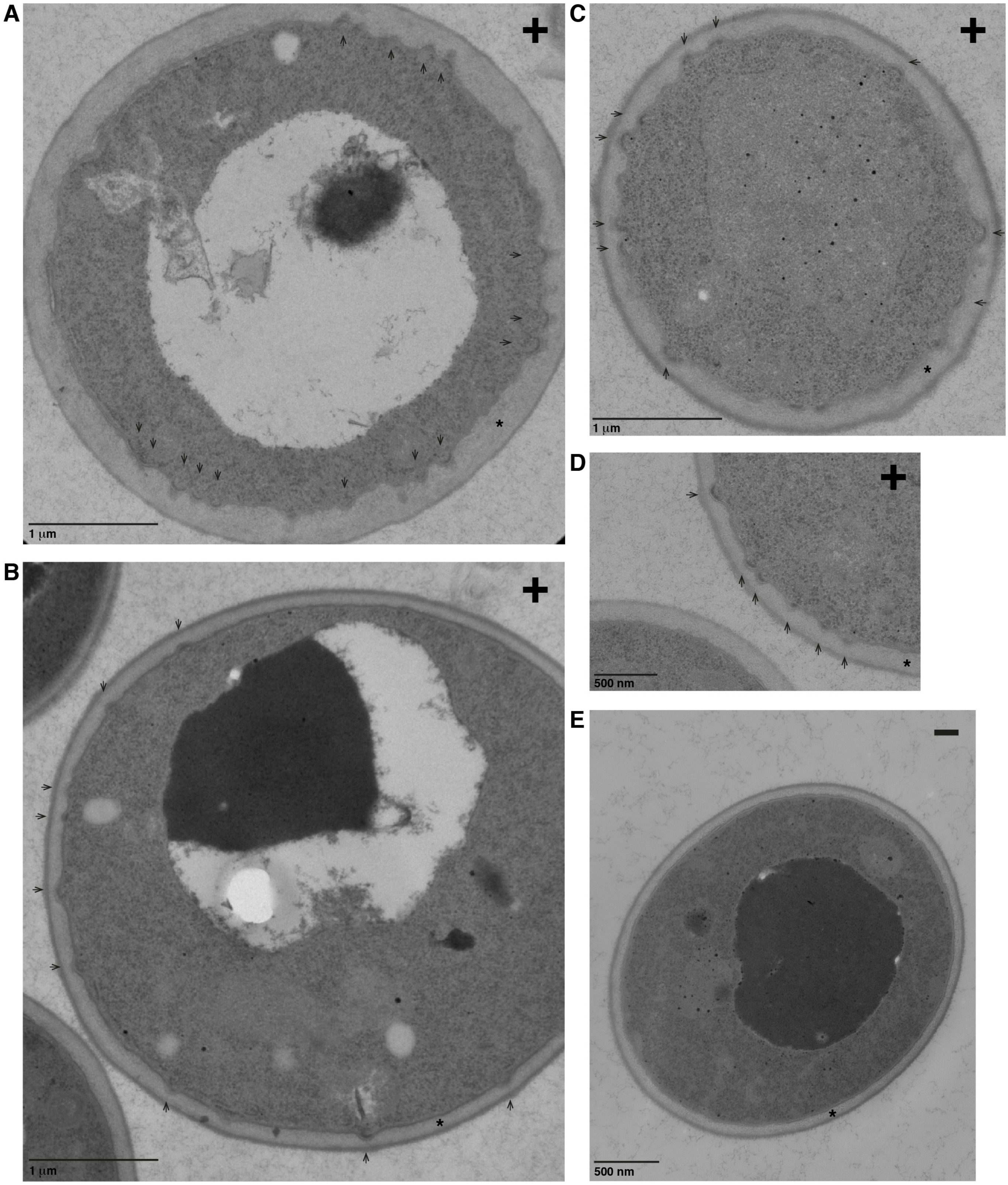

Fig. S5: WT yeast cells expressing Gag-GFP from a 2μ vector with MET3 promoter (+, **A.-D.**) or carrying the empty vector (-, **E**) were embedded in Epon. Sections were analyzed by EM, exhibiting buds (arrows) at the PM in presence

of Gag-GFP. Cells were grown in medium containing 20 mg/l methionine to repress the promoter. To induce the promoter, cells were shifted to medium lacking methionine for 6 h. The sections are derived from one experiment that is independent of the experiments shown in Fig. S4.

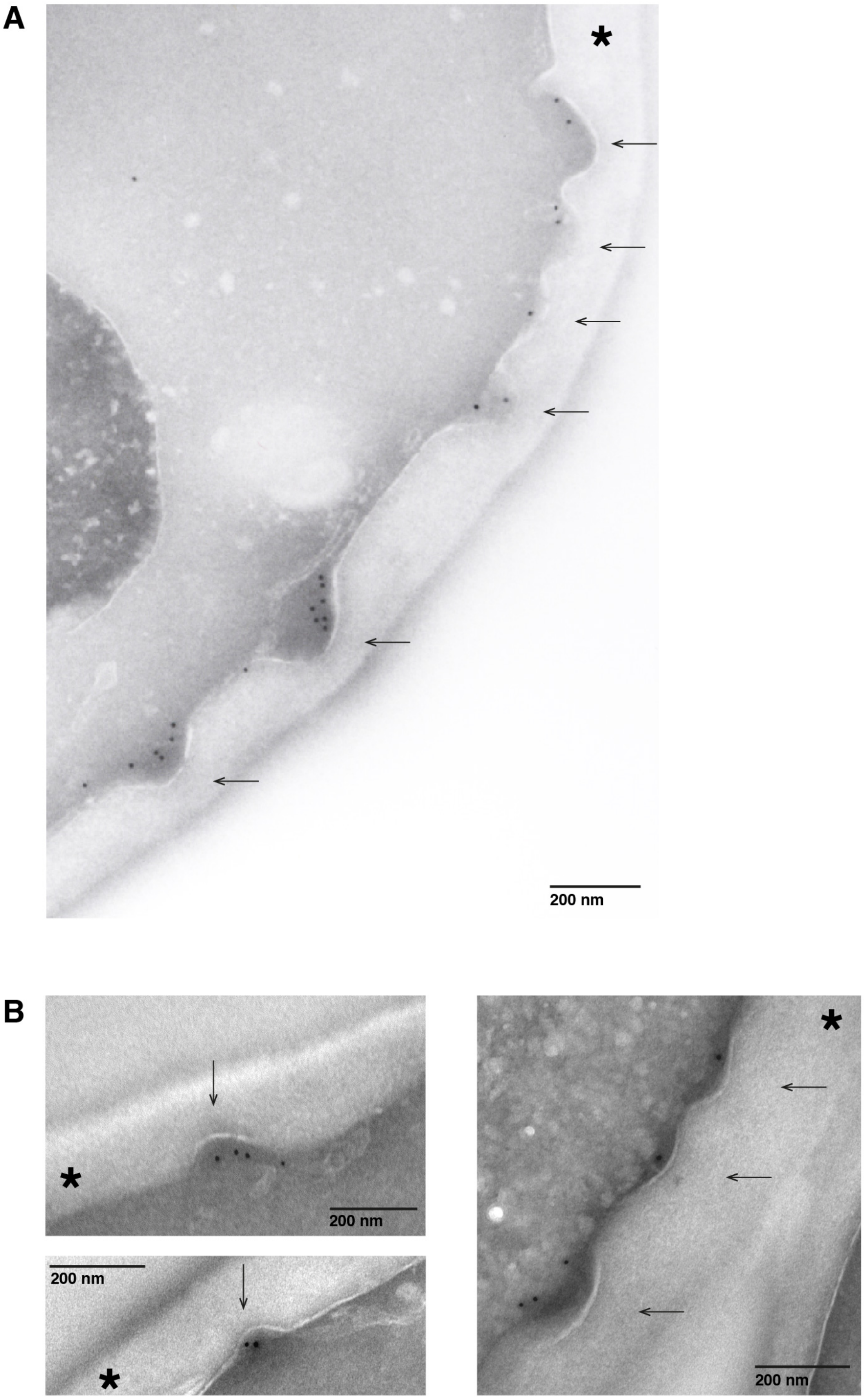

Fig. S6: PM deformation (arrows) induced by Gag-GFP expression was analyzed by EM. Cryosections of WT yeast cells (SUB62) expressing Gag-GFP from a 2μ vector with PGK promoter (**A.**) or of WT cells (YWO1) expressing Gag-GFP from a 2μ vector with MET3 promoter induced for 6 h (**B.**) were prepared. GFP was labeled with immunogold. Asterisks indicate the cell wall. A. is also shown in Fig. 1G.

Gag-GFP expression from the inducible MET3 promoter

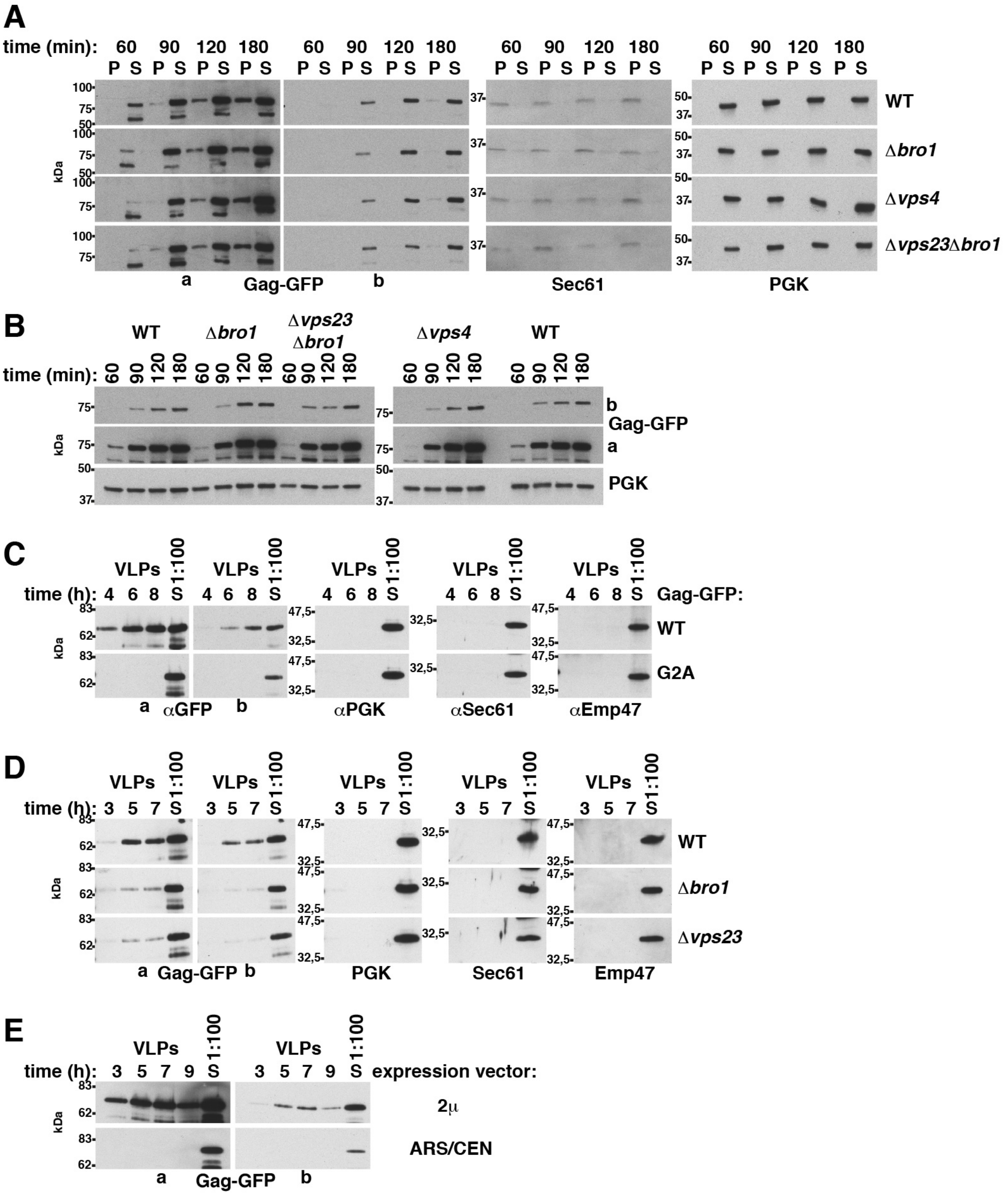

Gag-GFP expression from the constitutive PGK promoter

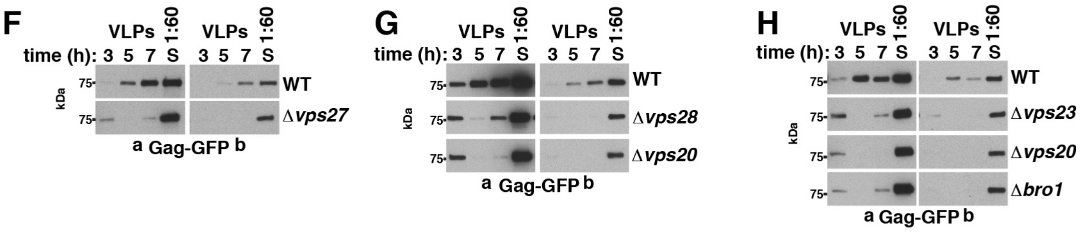

Fig. S7: **A. and B.** Gag-GFP-membrane binding after MET3-promoter induction was analyzed, showing that ESCRT deletion does not impair Gag-membrane binding. WT cells or the indicated ESCRT mutants carrying a Gag-GFP 2μ expression vector with MET3 promoter were grown in medium containing 20 mg/ml methionine to repress the promoter. To induce the promoter, cells were shifted to medium lacking methionine. After 60, 90, 120, and 360 min cell extracts (**B**) were prepared and separated by centrifugation at 25,000*g* into a membrane-containing sediment (P) and a cytosol-containing supernatant (S) (**A**). The samples were analyzed by immunoblotting with anti-GFP

antibodies. Sec61 (ER-membrane protein) and PGK (cytosolic protein) served as references. **C. and D.** Gag-GFP release from yeast spheroplasts was analyzed by immunoblotting of high-speed centrifugation sediments derived from the incubation medium with anti-GFP antibodies (VLPs). GFP-tagged Gag or Gag(G2A) was expressed from a 2µ vector with MET3 promoter in the WT or the indicated mutants. The promoter was induced when spheroplast preparation started. Immunoblots with antibodies detecting PGK (cytosolic protein), Sec61 (ER-membrane protein), or Emp47 (Golgi and COPII membrane protein) served as control for specific Gag release. S: lysate of spheroplasts prepared at the final VLP harvest. **C.** Showing myristoylation-dependent release. The first VLP harvest after 2-h incubation is not shown. **D.** Showing ESCRT-dependent release. **E.** Same as above except that Gag-GFP was expressed from a 2µ vector or an ARS/CEN vector with MET3 promoter, showing that VLPs cannot be harvested from spheroplasts expressing Gag-GFP from the ARS/CEN vector. The promoter was induced during yeast cultivation. **F.-H.** Same as above except that Gag-GFP was expressed from a 2µ vector with PGK promoter in the WT or the indicated mutants, showing ESCRT-dependent release. **A.-H.** Long (a) and short (b) exposures are shown.

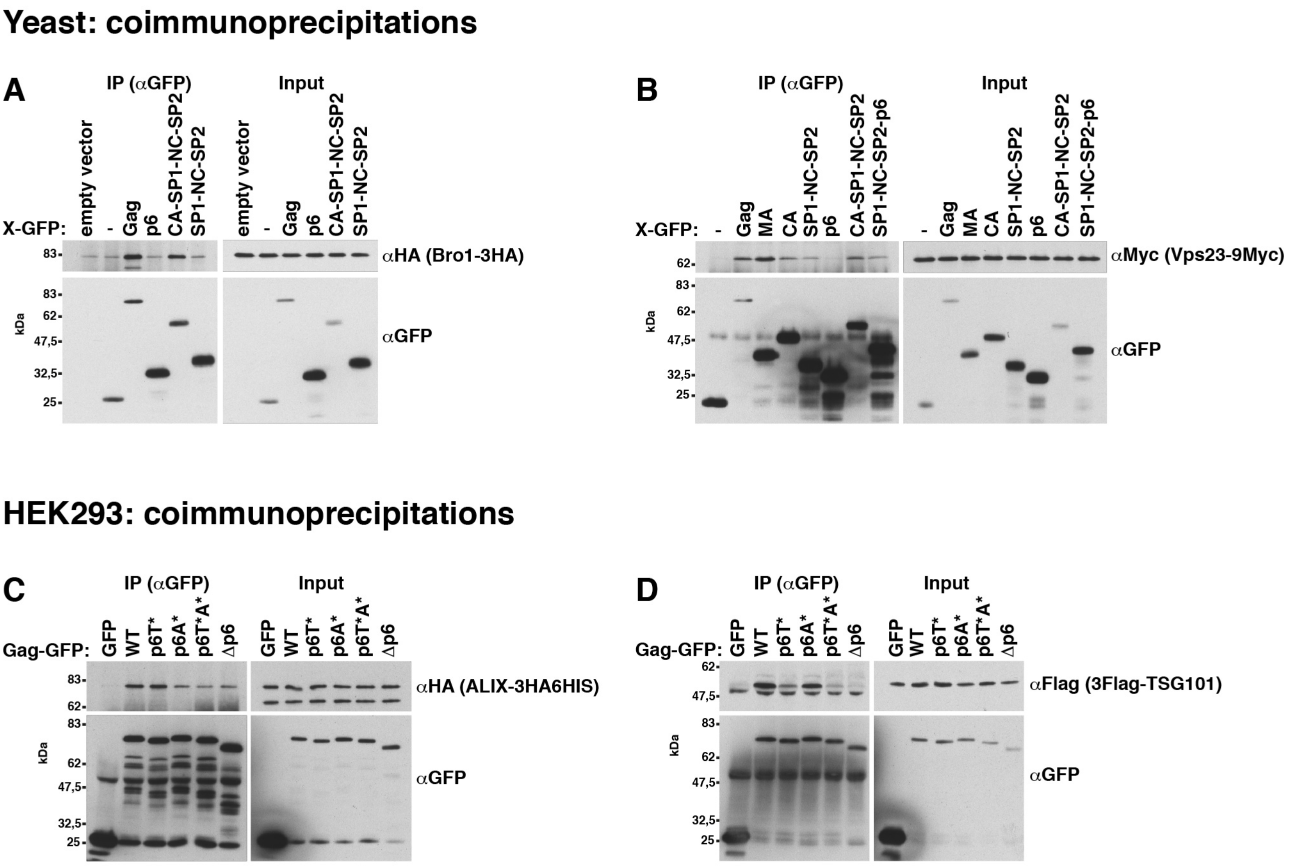

Fig. S8: A. and B. Coimmunoprecipitation experiments with GFP-tagged Gag or Gag fragments expressed from a 2µ vector with induced MET3 promoter in yeast strains carrying genomically epitope-tagged Bro1 or Vps23. Gag or Gag fragments were immunoprecipitated with anti-GFP antibodies and coimmunoprecipitated Bro1 or Vps23 was detected by immunoblotting with antibodies against the epitope tag. MA (aa 1-132), CA (aa 133-363), p6 (aa 448–500), CA-SP1-NC-SP2 (aa 133–447), SP1-NC-SP2 (aa 364-447), SP1-NC-SP2-p6 (aa 364–500). **A**. Showing that Bro1 binds to CA. In the presence of 400 mM NaCl. **B.** In the presence of 150 mM NaCl, showing that Vps23 coprecipitates with an NC-containing fragment under these salt conditions. **C. and D.** Coimmunoprecipitations of epitope-tagged ALIX or TSG101 with GFP-tagged Gag versions expressed from CMV-promoter vectors in HEK293 cells, showing that ALIX binding to Gag is reduced but not prevented by p6 deletion. TSG101 binding to Gag-GFP more strongly depends on p6. GFP-tagged proteins were immunoprecipitated with anti-GFP antibodies in the presence of 400 mM NaCl and coimmunopreciptated ALIX or TSG101 was detected with antibodies recognizing the epitope tag.

Yeast: GST-pull-downs

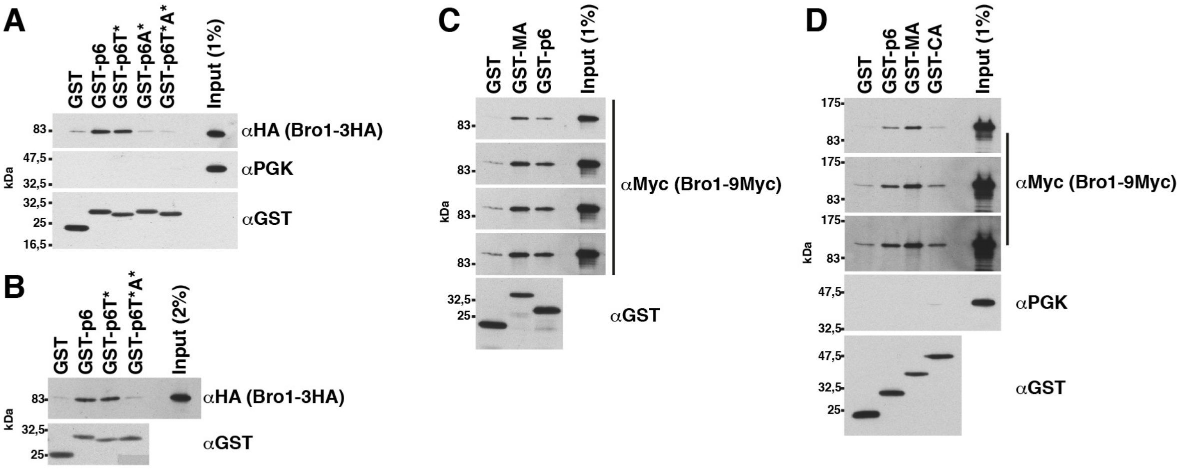

Yeast: coimmunoprecipitations

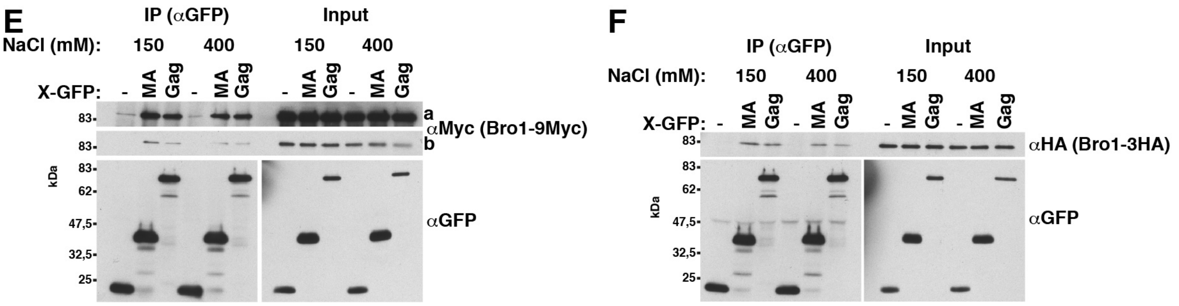

HEK293: GST-pull-downs

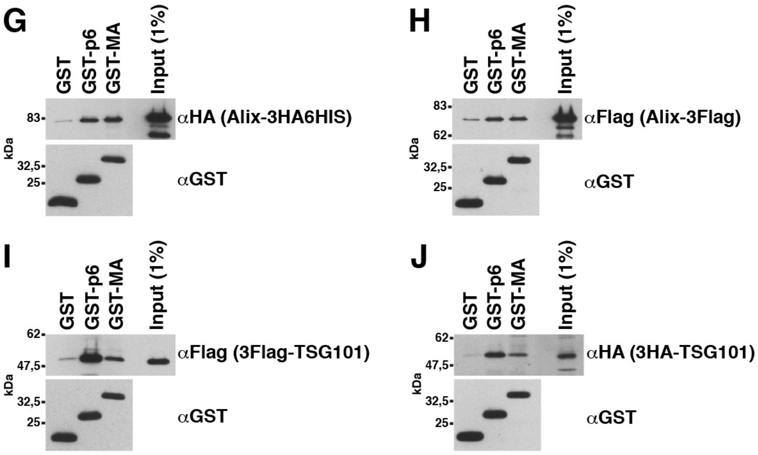

Fig. S9: Binding assays comparing different epitope tags. **A.–D.** GST-tagged Gag fragments (MA (aa 1-132), CA (aa 133-363), p6 (aa 448-500)) expressed in *E.coli* were bound to Glutathione Sepharose and incubated with extract of yeast cells carrying genomically epitope-tagged Bro1. The binding buffer contained 400 mM NaCl. Bead-bound proteins were analyzed by immunoblotting with the indicated antibodies. PGK served as control, showing specific Bro1 binding. P6T*: mutated TSG101 binding site, p6A*: mutated ALIX binding site. **A.** Fig. 3I shown for comparison. **D.** Fig. 3H with additional exposures shown for comparison. **E.–F.** Gag-GFP or MA-GFP was expressed from a 2μ vector with induced MET3 promoter in yeast strains carrying genomically epitope-tagged Bro1. GFP-

tagged proteins were immunoprecipitated with anti-GFP antibodies and coimmunoprecipitated Bro1 was detected by immunoblotting with anti-HA or anti-Myc antibodies. A long (a) and a short (b) exposure are shown. **G.–J.** GST-tagged MA or p6 expressed in *E.coli* was bound to Glutathione Sepharose and incubated with extract of HEK293 cells expressing epitope-tagged ALIX or TSG101 from a CMV-promoter vector in the presence of 150 mM NaCl. Bead-bound proteins were analyzed by immunoblotting with the indicated antibodies.

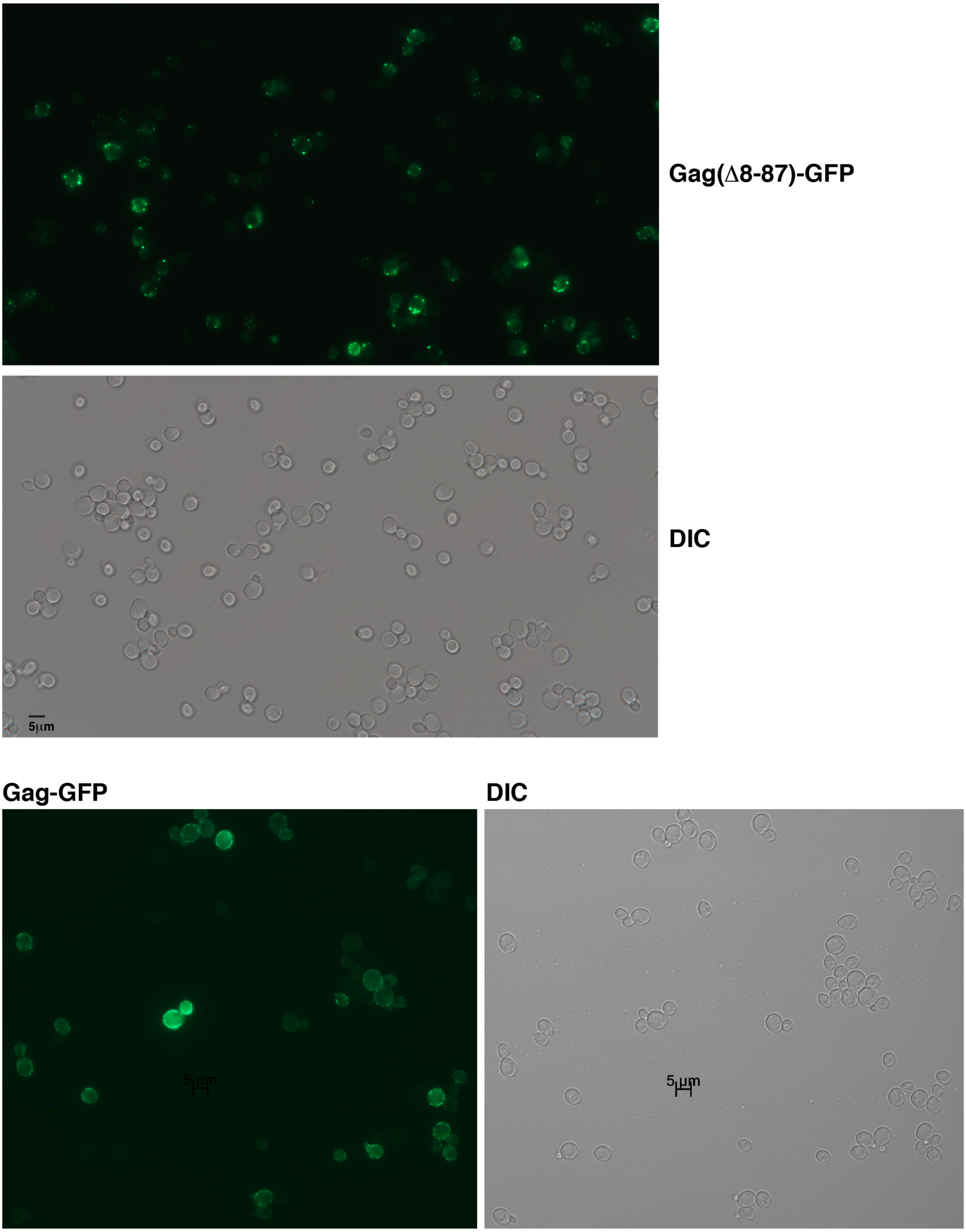

Fig. S10: Gag(Δ8-87)-GFP or Gag-GFP expressed from a 2μ vector with PGK promoter in WT yeast cells was analyzed by fluorescence microscopy. Compared to Gag-GFP, Gag(Δ8-87)-GFP forms fewer structures at the PM with larger diameters. DIC: differential interference contrast. The Gag-GFP image is also shown in Fig. S2.

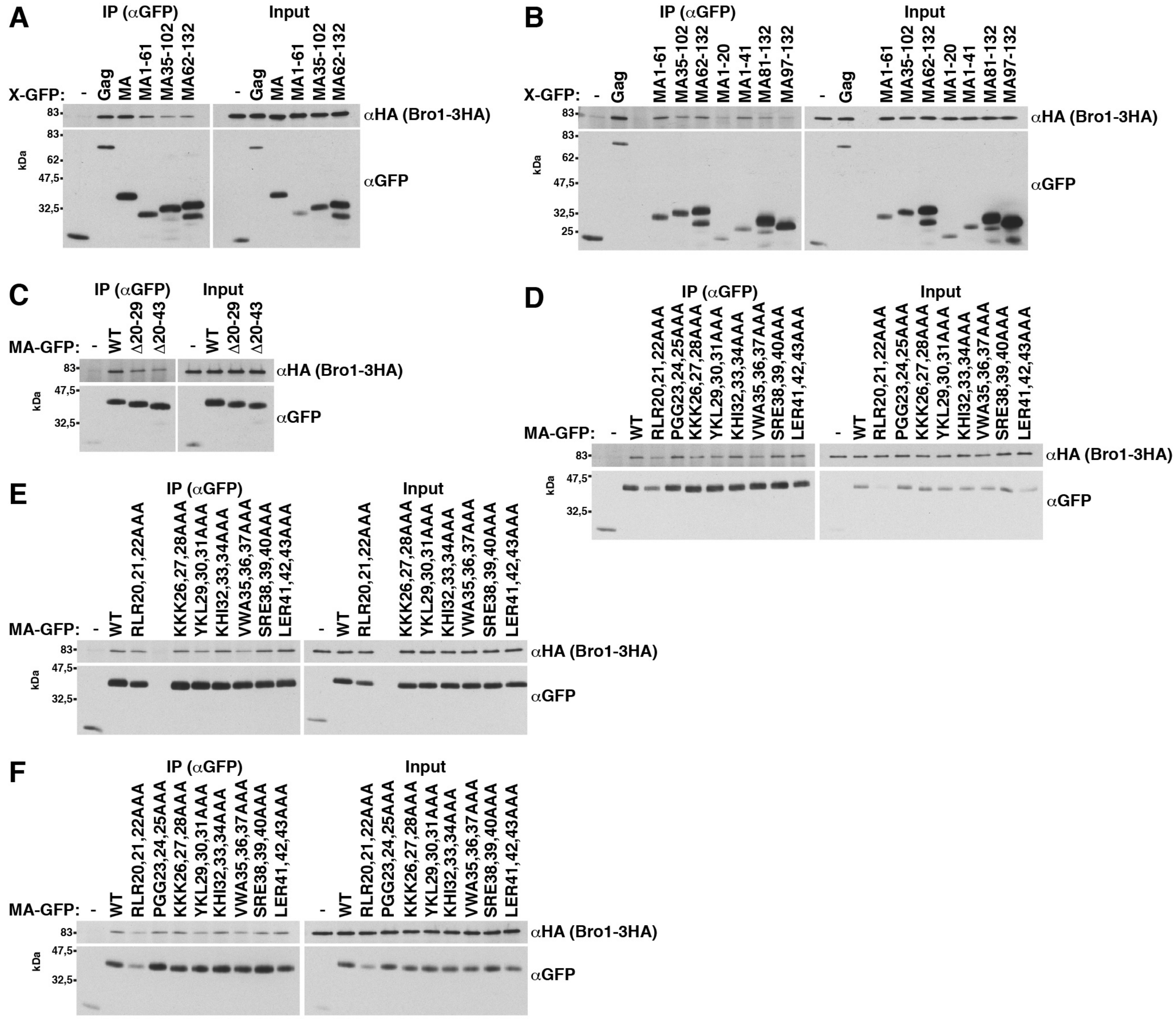

Fig. S11: Examples of coimmunoprecipitation experiments that were done to characterize the MA-Bro1 interaction. GFP-tagged Gag, Gag fragments, or MA (aa 1-132) versions were expressed from a 2μ vector with induced MET3 promoter in yeast cells carrying genomically 3HA-tagged Bro1. GFP-tagged proteins or peptides were immunoprecipitated with anti-GFP antibodies in the presence of 400 mM NaCl and coimmunoprecipitated Bro1 was detected by immunoblotting with anti-HA antibodies. **A.-C.** With MA fragments or internal-deletion mutants, indicating that helix-2 (aa 30-43) and the strand loop between helix-1 and 2 (aa 20-29) might be involved in the MA-Bro1 interaction. Secondary structural-element assignments are derived from the MA x-ray structure (11). **D.-F.** AAA-scanning mutagenesis for MA residues 20 to 43, showing that YKL29,30,31AAA and VWA35,36,37AAA reduce the MA-Bro1 interaction. As the effects are weak, three experiments are shown to prove that the results are reproducible.

Yeast: coimmunoprecipitations

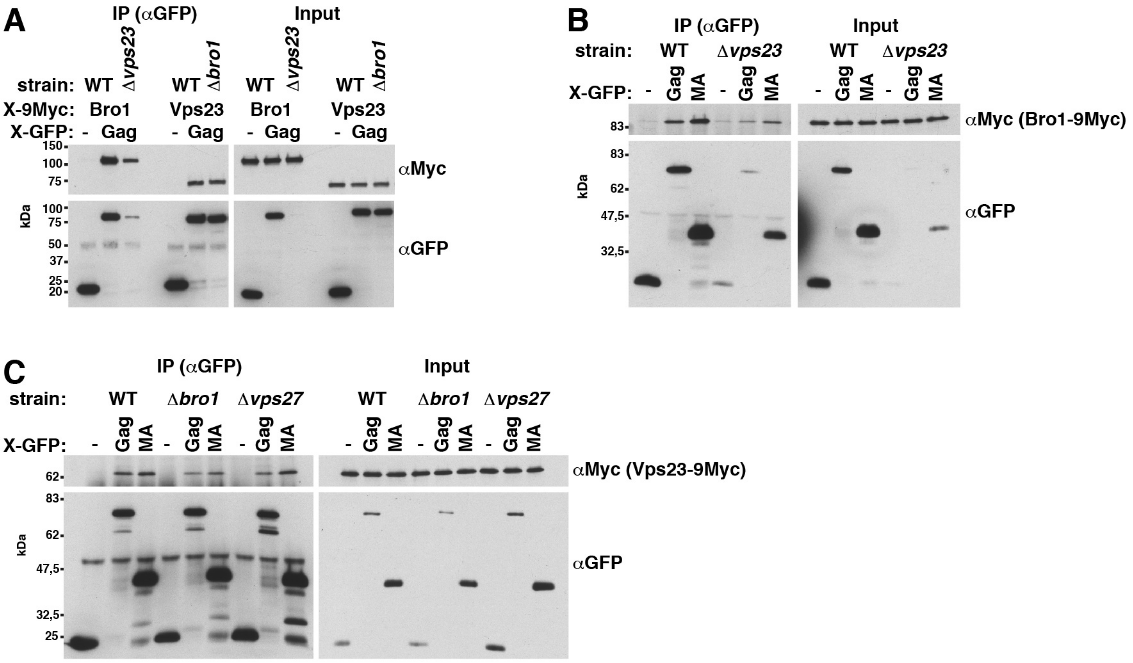

Yeast: GST-pull-downs

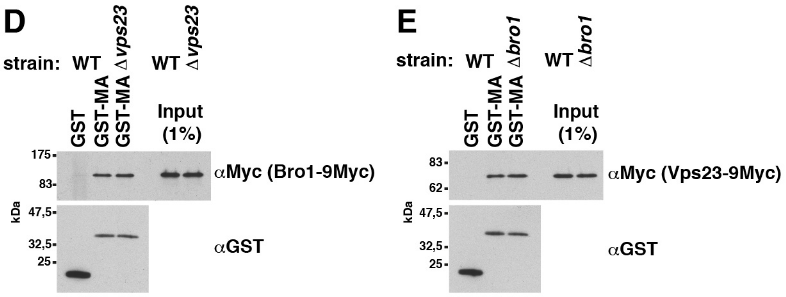

Fig. S12: A.-C. Coimmunoprecipitation experiments with GFP-tagged Gag or MA (aa 1-132) expressed from a 2μ vector with induced MET3 promoter in yeast strains carrying genomically 9Myc-tagged Vps23 or Bro1. Gag or MA was immunoprecipitated with anti-GFP antibodies and coimmunoprecipitated Vps23 or Bro1 was detected by immunoblotting with anti-Myc antibodies in the presence of 400 mM NaCl. **A.** With WT yeast cells or the indicated mutants, showing that Vps23 coimmunoprecipitated with Gag independent of Bro1. Bro1 coimmunoprecipitated with Gag expressed in a *Δvps23* mutant. Because Gag expression was reduced in this strain, the coimmunoprecipitated Bro1 amount cannot be directly compared with that precipitated from the WT strain. **B.** With WT yeast cells or a *Δvps23* mutant, showing that Bro1 coimmunoprecipitated with MA in absence of Vps23. Same problem as in A. **C.** With WT yeast cells or the indicated mutants, showing that Vps23 coimmunoprecipitated with Gag and MA in absence of Bro1 or Vps27, another ubiquitin-binding ESCRT protein. **D. and E.** Pull-down experiments, showing that genomically 9Myc-tagged Bro1 or Vps23 bound to GST-MA expressed in *E. coli* while VPS23 or BRO1 was deleted. GST-MA (aa 1-132) was bound to Glutathione Sepharose and incubated with yeast extract in the presence of 150 mM NaCl. Bead-bound proteins were analyzed by immunoblotting with the indicated antibodies.

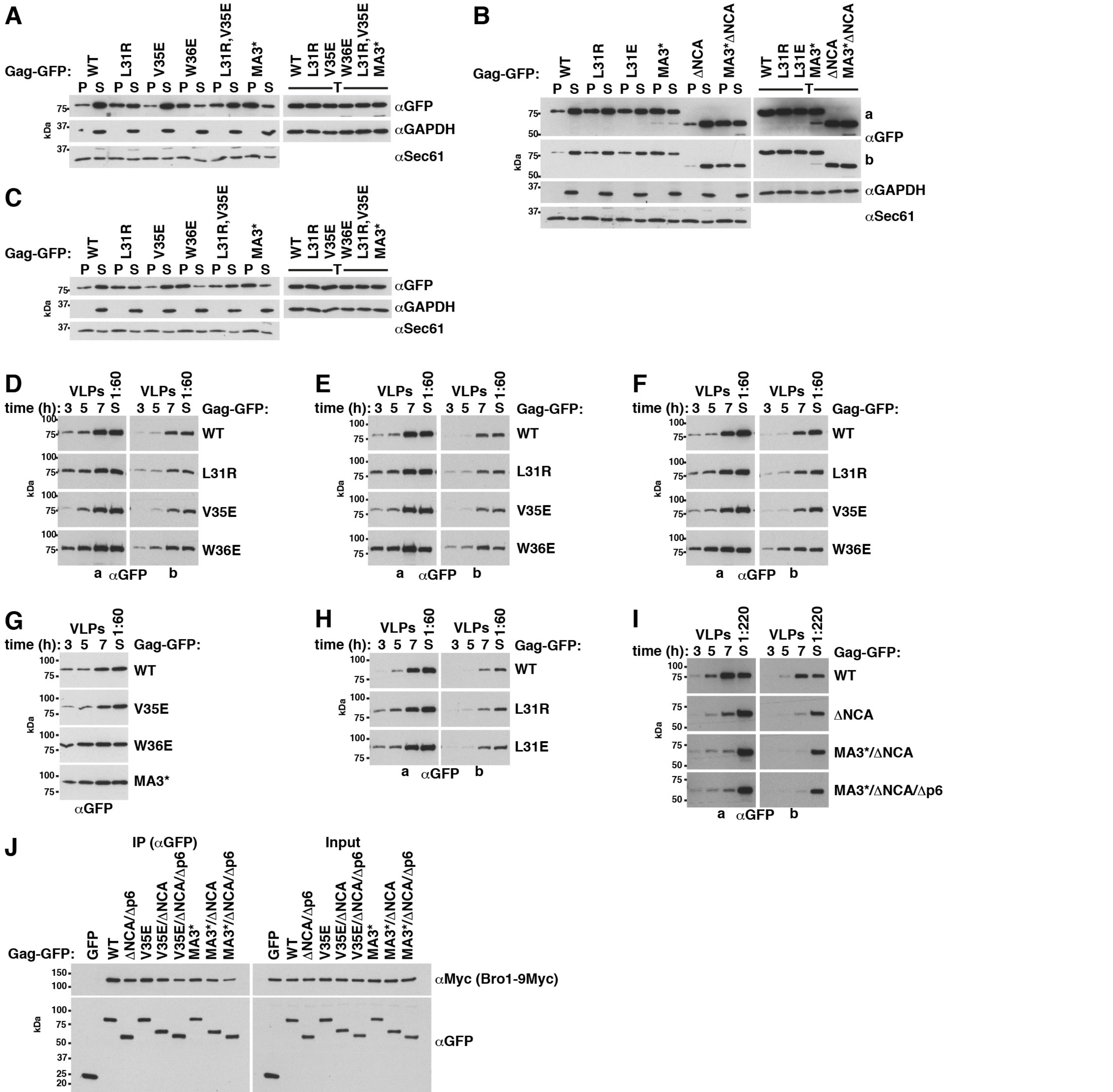

Fig. S13: A.-C. Membrane-containing 25,000*g* pellets (P) and cytosol-containing supernatants (S) derived from cell extracts (T) were analyzed by immunoblotting with the indicated antibodies, showing that L31 or W36 mutations increase Gag-membrane binding and that the ΔNCA mutation does not reduce Gag-membrane association. The cytosolic protein GAPDH and the integral ER-membrane protein Sec61 served as references. **D.-I.** Release of Gag-GFP versions from WT yeast spheroplasts was analyzed by immunoblotting of high-speed centrifugation sediments derived from the incubation medium with anti-GFP antibodies (VLPs). S: lysate of spheroplasts prepared at the final VLP harvest. **D.-H.** Showing that MA mutations in L31 and W36 increase Gag release. **I.** Showing that ΔNCA reduces Gag release, is dominant over MA3*, and that p6 deletion does not further reduce Gag(MA3*/ΔNCA) release. **A.-I.** Gag-GFP versions were expressed from a 2μ vector with PGK promoter in WT yeast. Long (a) and short (b) exposures are shown. **J.** Coimmunoprecipitation experiment with Gag-GFP versions expressed from a 2μ vector with induced MET3 promoter in yeast cells carrying genomically 9Myc-tagged Bro1, showing that although V35E does not reduce the Gag-Bro1 coimmunoprecipitation, the Bro1 amount that coimmunoprecipitates with

Gag(V35E/ΔNCA/Δp6)-GFP is reduced compared to Gag(ΔNCA/Δp6)-GFP. Gag was immunoprecipitated with anti-GFP antibodies in the presence of 400 mM NaCl and coimmunoprecipitated Bro1 was detected by immunoblotting with anti-Myc antibodies.

Gag(MA3*)-GFP

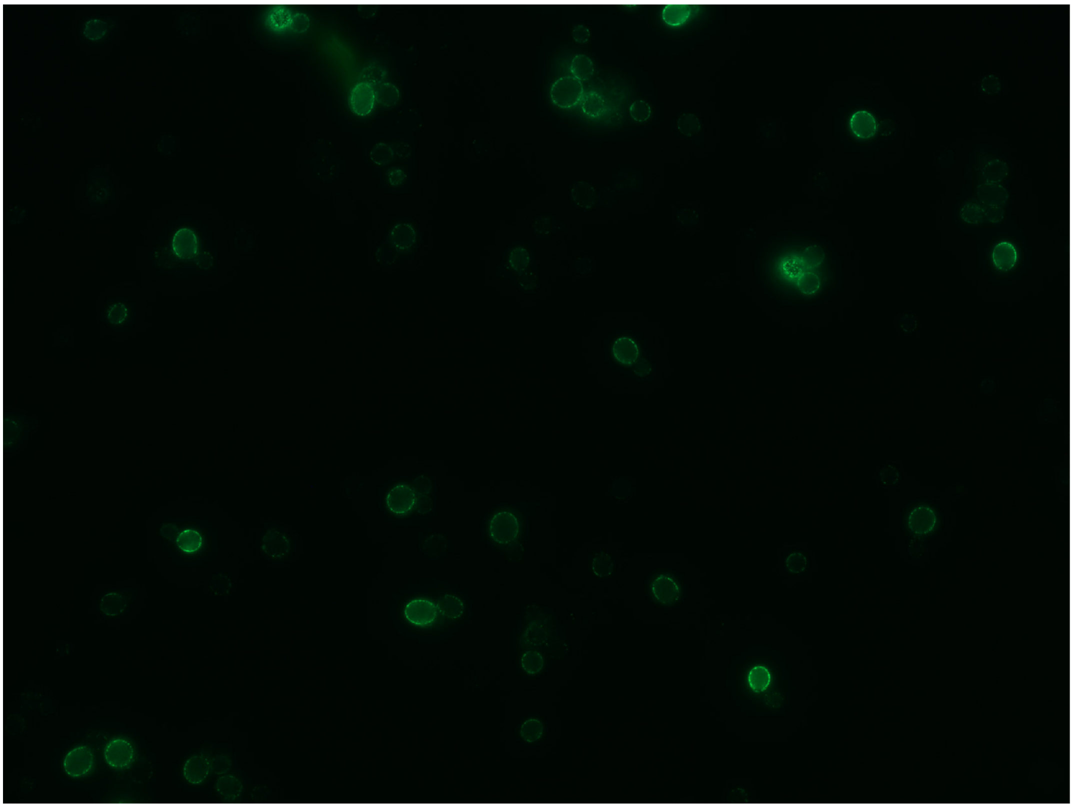

DIC

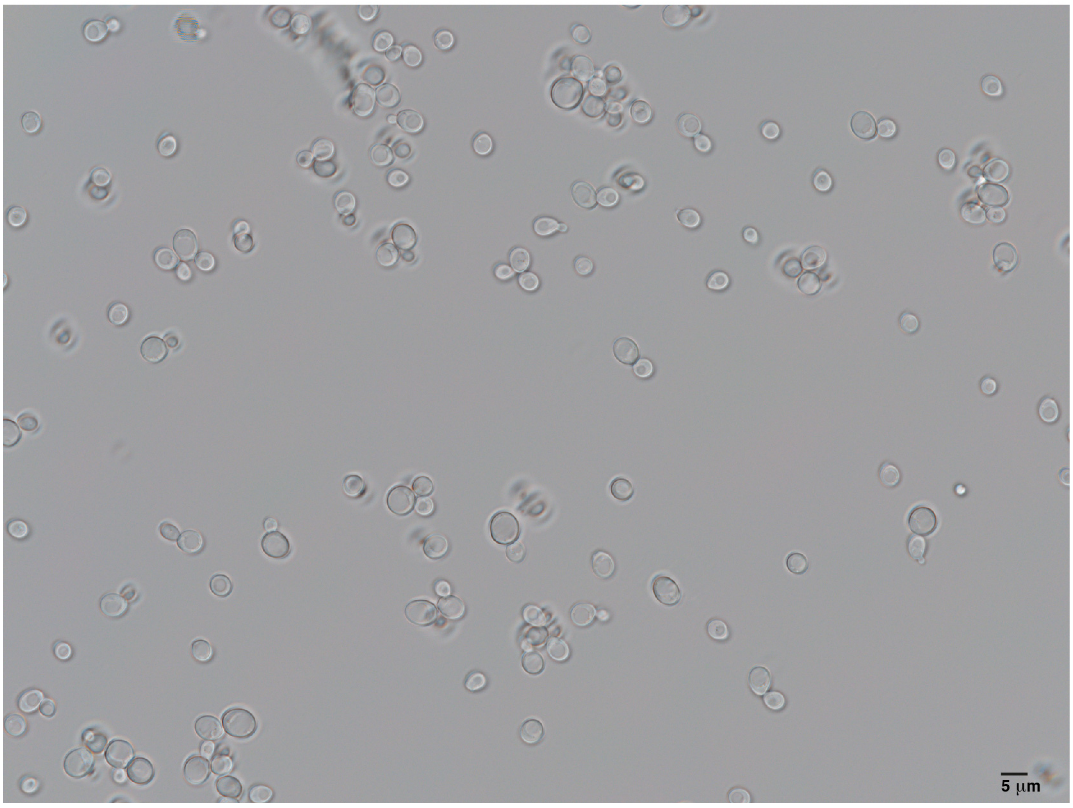

Fig. S14: Gag(MA3*)-GFP expressed from a 2µ vector with PGK promoter in WT yeast cells was analyzed by fluorescence microscopy, showing that Gag(MA3*)-GFP forms punctate structures at the PM similar to Gag-GFP (Fig. S1, S2) and does not form aggregates. DIC: differential interference contrast.

Gag(ΔNCA)-GFP

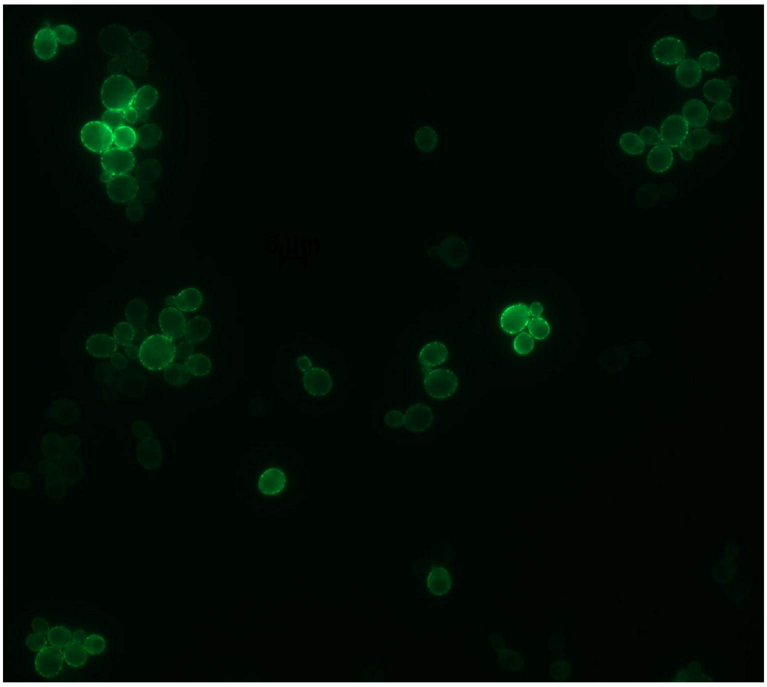

DIC

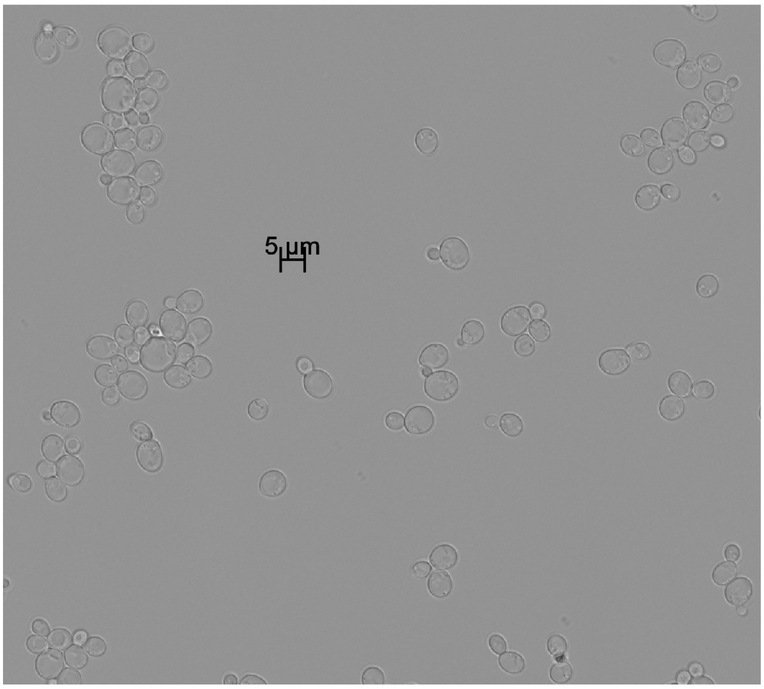

Fig. S15: Gag(ΔNCA)-GFP expressed from a 2µ vector with PGK promoter in WT yeast cells was analyzed by fluorescence microscopy, showing that Gag(ΔNCA)-GFP forms punctate structures at the PM similar to Gag-GFP (Fig. S1 and S2) and does not form aggregates. DIC: differential interference contrast.

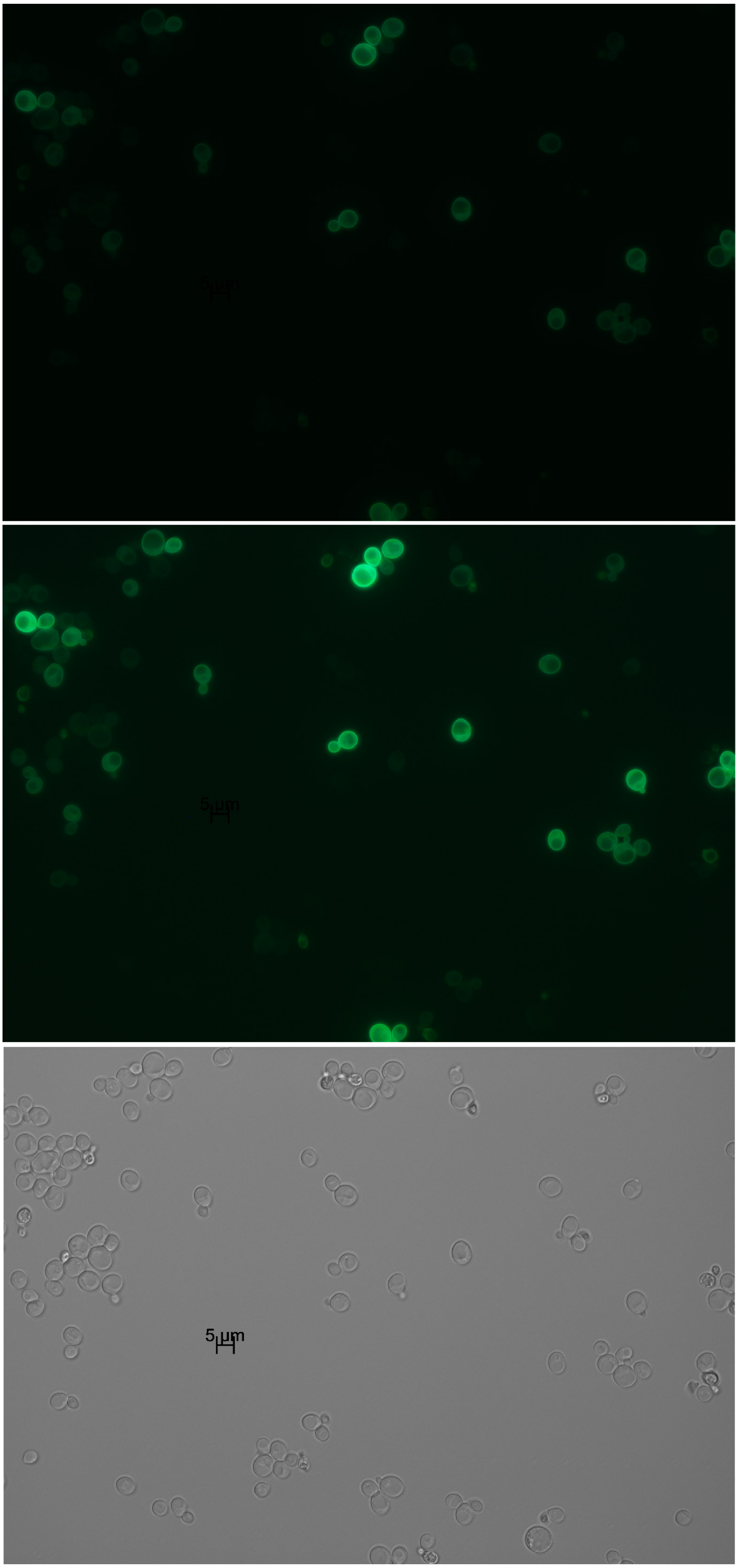

a

MA3*-GFP

b

DIC

Fig. S16: MA3*-GFP expressed from a 2μ vector with PGK promoter in WT yeast cells was analyzed by fluorescence microscopy, showing PM rim staining. DIC: differential interference contrast, b: enhanced brightness.

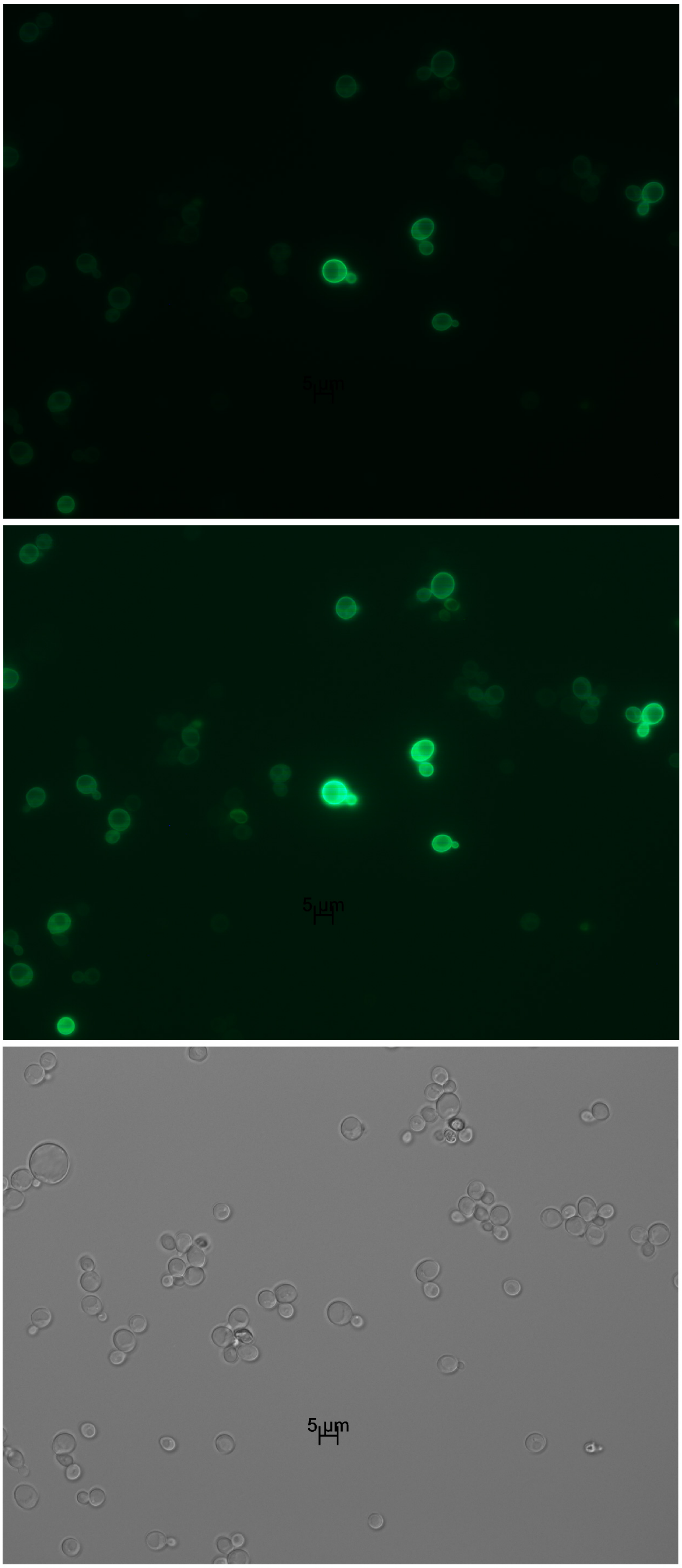

a

MA3*-GFP

b

DIC

Fig. S17: MA3*-GFP expressed from a 2µ vector with PGK promoter in WT yeast cells was analyzed by fluorescence microscopy, showing PM rim staining. DIC: differential interference contrast, b: enhanced brightness.

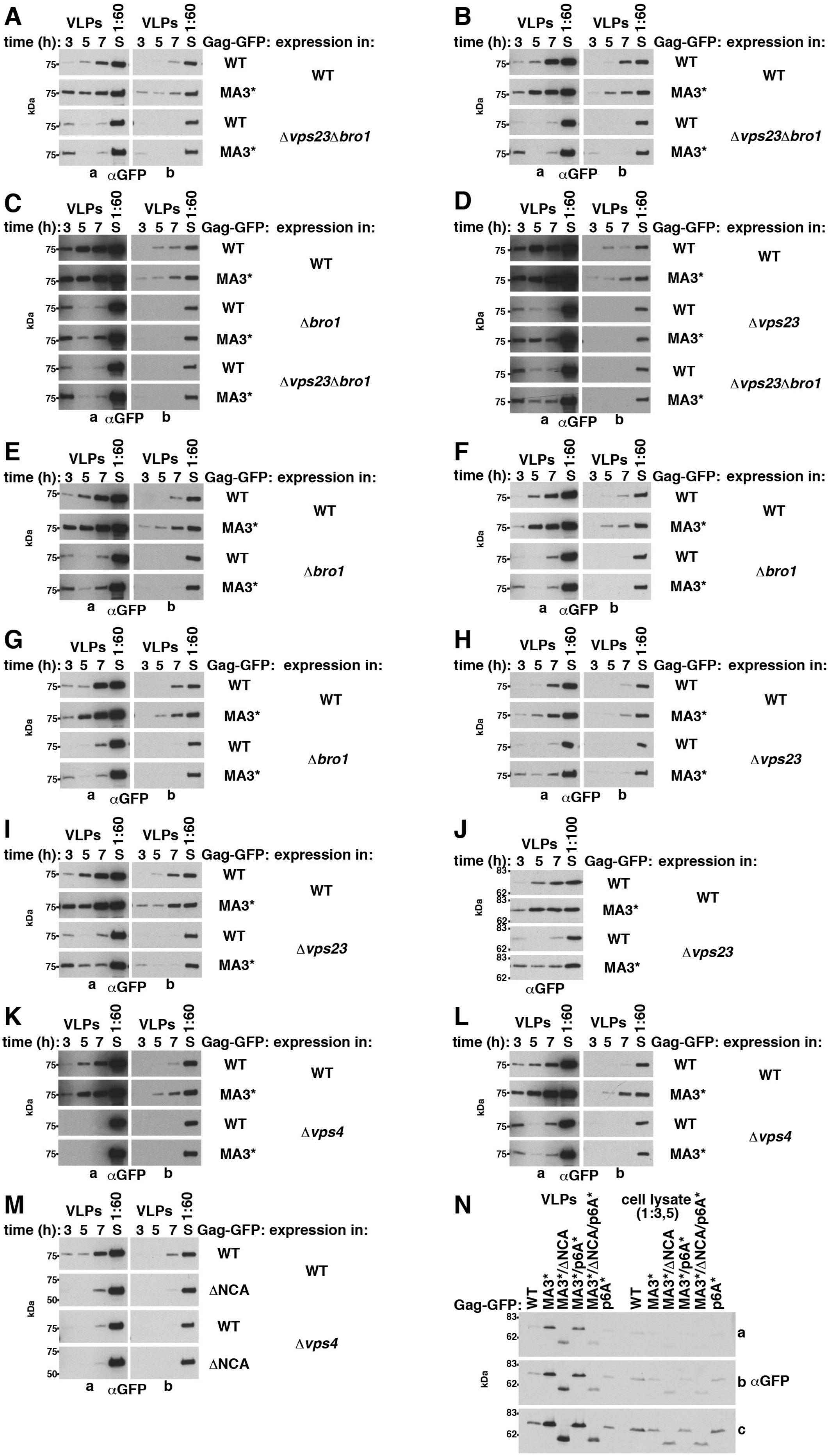
A
B
C
D
E
F
G
H
I
J
K
L
M
N
VLPs
1:60
1:100
time (h): 3 5 7 S
Gag-GFP: expression in:
WT
MA3*
ΔNCA
Δvps23Δbro1
Δbro1
Δvps23
Δvps4
kDa
75
83
62
50
a
b
c
αGFP
cell lysate (1:3,5)
MA3*/ΔNCA
MA3*/p6A*
MA3*/ΔNCA/p6A*
p6A*

Fig. 18: Gag-GFP release from yeast spheroplasts was analyzed by immunoblotting of high-speed centrifugation sediments derived from the incubation medium with anti-GFP antibodies (VLPs). Gag-GFP versions were expressed from a 2µ vector with PGK promoter in the WT or the indicated mutants. S: lysate of spheroplasts prepared at the final VLP harvest. Long (a) and short (b) exposures are shown. **A.-D.** *Δvps23Δbro1* nearly abolished the MA3* effect after 5 and 7 h of incubation. **C., E.-G.** Gag(MA3*)-GFP release from *Δbro1* spheroplasts was increased compared to Gag-GFP in 2 experiments (C. and E.), whereas the MA3* effect was abolished in two other experiments (F. and G.). **D., H. –J.** Gag(MA3*)-GFP release from *Δvps23* spheroplasts was increased compared to Gag-GFP. **K. and L.** *Δvps4* abolished the MA3* effect. **M.** Gag(ΔNCA)-GFP release from *Δvps4* spheroplasts was slightly decreased compared to Gag-GFP. **N.** Gag-GFP release from HEK293 cells 2 d after transfection with CMV-promoter expression vectors for the indicated Gag versions. VLPs were harvested from the culture medium and cell lysates were prepared. Gag-GFP was detected by immunoblotting with anti-GFP antibodies. Several exposures (a,b,c) are shown. The MA3* mutation increased Gag-GFP release. A combination of ΔNCA and mutation of the ALIX binding site in p6 (p6A*) abrogated this increase, whereas an isolated p6A* mutation did not impair Gag-GFP release.

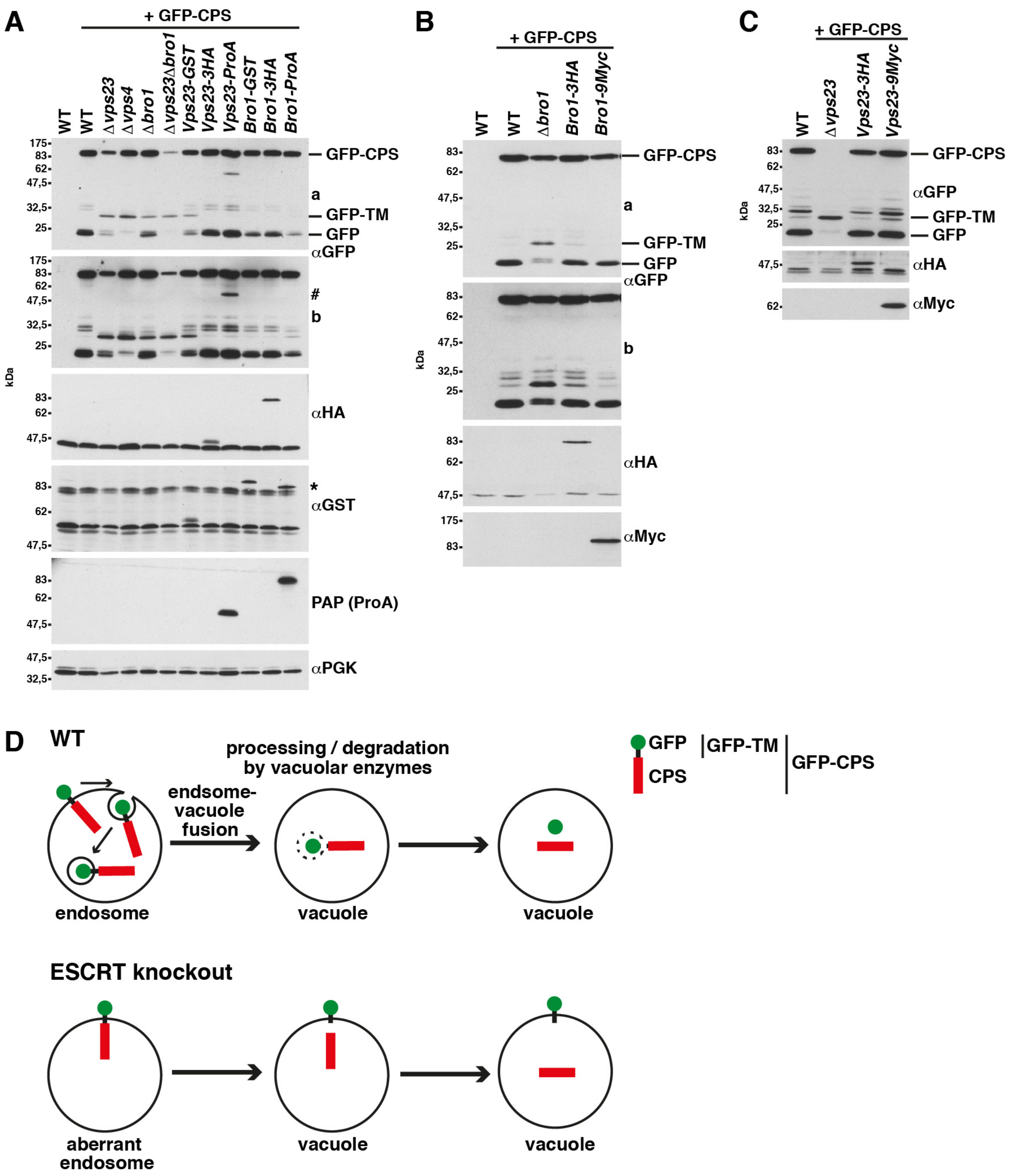

Fig. S19: Bro1-3HA, Bro-9Myc, and Vps23-9Myc are functional in the CPS-transport assay. **A.–C.** The vacuolar processing of N-terminally GFP-tagged carboxypeptidase S (GFP-CPS) was analyzed in yeast strains carrying genomically epitope-tagged ESCRT proteins (31, 129). **D.** CPS is a soluble vacuolar enzyme that is synthesized as a precursor type II transmembrane protein, transported to the vacuolar interior by the MVB pathway, and cleaved from its transmembrane anchor by vacuolar hydrolases. In GFP-CPS transport, the GFP moiety is initially located on the cytosolic side of the endosomal membrane, GFP-CPS is sorted into MVB vesicles and transported into the endosomal interior. After endosome-vacuole fusion, vacuolar enzymes degrade the vesicles and release free GFP and CPS. In ESCRT knockout mutants, MVB vesicle formation is impaired and GFP-CPS accumulates in the limiting endosomal and vacuolar membranes. After proteolytic processing, GFP remains connected to the short cytosolic and transmembrane domain of CPS (GFP-TM). **A.–C.** Lysate of WT cells, ESCRT knockout mutants, or strains with genomically epitope-tagged Bro1 or Vps23 carrying a GFP-CPS expression plasmid were analyzed by immunoblotting with the indicated antibodies. PGK served as loading control. Short (a) and long (b) exposures are shown. #, *: signal of Protein A (ProA) reactivity with antibodies used for the detection of other epitope tags. PAP: peroxidase-anti-peroxidase complex.

Yeast cells were disrupted with glass beads in 50 mM Tris (pH 7.5) and 1% SDS containing protease inhibitors. The lysates were cleared by centrifugation (10 min, 16,000*g*).

Table S1: yeast strains used in this study.

Strain	Genotype	Reference
YWO1	*MATα, trp-1(am), his3-Δ200, ura3-52, lys2-801, leu2-3, -112*	Ref. 136
YBM87	*MATα,* ***vps23::HIS3****, trp-1(am), his3-Δ200, ura3-52, lys2-801, leu2-3, -112*	this study
YBM88	*MATα,* ***bro1::KanMX6****, trp-1(am), his3-Δ200, ura3-52, lys2-801, leu2-3, -112*	this study
YBM89	*MATα,* ***vps23::HIS3, bro1::KanMX6****, trp-1(am), his3-Δ200, ura3-52, lys2-801, leu2-3, -112*	this study
YBM90	*MATα,* ***vps4::HIS3****, trp-1(am), his3-Δ200, ura3-52, lys2-801, leu2-3, -112*	this study
YBM91	*MATα,* ***vps20::HIS3****, trp-1(am), his3-Δ200, ura3-52, lys2-801, leu2-3, -112*	this study
YBM92	*MATα,* ***vps27::HIS3****, trp-1(am), his3-Δ200, ura3-52, lys2-801, leu2-3, -112*	this study
YBM93	*MATα,* ***vps28::HIS3****, trp-1(am), his3-Δ200, ura3-52, lys2-801, leu2-3, -112*	this study
YBM94	*MATα,* ***Bro1-3HA:HIS3****, trp-1(am), his3-Δ200, ura3-52, lys2-801, leu2-3, -112*	this study
YBM95	*MATα,* ***Bro1-9Myc:TRP1****, trp-1(am), his3-Δ200, ura3-52, lys2-801, leu2-3, -112*	this study
YBM96	*MATα,* ***Vps23-9Myc:TRP1****, trp-1(am), his3-Δ200, ura3-52, lys2-801, leu2-3, -112*	this study
YBM97	*MATα,* ***Bro1-9Myc:TRP1****,* ***vps23::KanMX6,*** *trp-1(am), his3-Δ200, ura3-52, lys2-801, leu2-3, -112*	this study
YBM98	*MATα,* ***Vps23-9Myc:TRP1****,* ***bro1::KanMX6****, trp-1(am), his3-Δ200, ura3-52, lys2-801, leu2-3, -112*	this study
YBM99	*MATα,* ***Vps23-9Myc:TRP1****,* ***vps27::KanMX6****, trp-1(am), his3-Δ200, ura3-52, lys2-801, leu2-3, -112*	this study
SUB62	*MATa, trp1-1(am), his3-Δ200, ura3-52, lys2-801, leu2-3,112*	Ref. 137
